# *Ligilactobacillus salivarius* Strains Isolated From the Porcine Gut Modulate Innate Immune Responses in Epithelial Cells and Improve Protection Against Intestinal Viral-Bacterial Superinfection

**DOI:** 10.3389/fimmu.2021.652923

**Published:** 2021-06-07

**Authors:** Yuhki Indo, Shugo Kitahara, Mikado Tomokiyo, Shota Araki, Md. Aminul Islam, Binghui Zhou, Leonardo Albarracin, Ayako Miyazaki, Wakako Ikeda-Ohtsubo, Tomonori Nochi, Takato Takenouchi, Hirohide Uenishi, Hisashi Aso, Hideki Takahashi, Shoichiro Kurata, Julio Villena, Haruki Kitazawa

**Affiliations:** ^1^ Food and Feed Immunology Group, Laboratory of Animal Food Function, Graduate School of Agricultural Science, Tohoku University, Sendai, Japan; ^2^ Livestock Immunology Unit, International Education and Research Center for Food Agricultural Immunology (CFAI), Graduate School of Agricultural Science, Tohoku University, Sendai, Japan; ^3^ Department of Medicine, Faculty of Veterinary Science, Bangladesh Agricultural University, Mymensingh, Bangladesh; ^4^ Scientific Computing Laboratory, Computer Science Department, Faculty of Exact Sciences and Technology, National University of Tucuman, Tucuman, Argentina; ^5^ Laboratory of Immunobiotechnology, Reference Centre for Lactobacilli, (CERELA-CONICET), Tucuman, Argentina; ^6^ Viral Diseases and Epidemiology Research Division, National Institute of Animal Health, NARO, Tsukuba, Japan; ^7^ Laboratory of Functional Morphology, Graduate School of Agricultural Science, Tohoku University, Sendai, Japan; ^8^ Animal Bioregulation Unit, Division of Animal Sciences, Institute of Agrobiological Sciences, National Agriculture and Food Research Organization (NARO), Tsukuba, Ibaraki, Japan; ^9^ Laboratory of Animal Health Science, Graduate School of Agricultural Science, Tohoku University, Sendai, Japan; ^10^ Laboratory of Plant Pathology, Graduate School of Agricultural Science, Tohoku University, Sendai, Japan; ^11^ Plant Immunology Unit, International Education and Research Center for Food and Agricultural Immunology (CFAI), Graduate School of Agricultural Science, Tohoku University, Sendai, Japan; ^12^ Laboratory of Molecular Genetics, Graduate School of Pharmaceutical Sciences, Tohoku University, Sendai, Japan

**Keywords:** porcine intestinal epithelial cells, rotavirus infection, innate immunity, intestinal superinfection, lactobacilli

## Abstract

Previously, we constructed a library of *Ligilactobacillus salivarius* strains from the intestine of wakame-fed pigs and reported a strain-dependent capacity to modulate IFN-β expression in porcine intestinal epithelial (PIE) cells. In this work, we further characterized the immunomodulatory activities of *L. salivarius* strains from wakame-fed pigs by evaluating their ability to modulate TLR3- and TLR4-mediated innate immune responses in PIE cells. Two strains with a remarkable immunomodulatory potential were selected: *L. salivarius* FFIG35 and FFIG58. Both strains improved IFN-β, IFN-λ and antiviral factors expression in PIE cells after TLR3 activation, which correlated with an enhanced resistance to rotavirus infection. Moreover, a model of enterotoxigenic *E. coli* (ETEC)/rotavirus superinfection in PIE cells was developed. Cells were more susceptible to rotavirus infection when the challenge occurred in conjunction with ETEC compared to the virus alone. However, *L. salivarius* FFIG35 and FFIG58 maintained their ability to enhance IFN-β, IFN-λ and antiviral factors expression in PIE cells, and to reduce rotavirus replication in the context of superinfection. We also demonstrated that FFIG35 and FFIG58 strains regulated the immune response of PIE cells to rotavirus challenge or ETEC/rotavirus superinfection through the modulation of negative regulators of the TLR signaling pathway. *In vivo* studies performed in mice models confirmed the ability of *L. salivarius* FFIG58 to beneficially modulate the innate immune response and protect against ETEC infection. The results of this work contribute to the understanding of beneficial lactobacilli interactions with epithelial cells and allow us to hypothesize that the FFIG35 or FFIG58 strains could be used for the development of highly efficient functional feed to improve immune health status and reduce the severity of intestinal infections and superinfections in weaned piglets.

## Introduction

Viruses from the family *Reoviridae* are non-enveloped viruses with an icosahedral capsid and a segmented genome of double-stranded RNA (dsRNA) molecules. Among this family of viruses, rotaviruses and reoviruses are capable of infecting pigs (1, 2). Both clinical and subclinical rotavirus infections have been documented in pigs and it has been established that young animals are more susceptible to severe disease when compared to immunocompetent adults (3, 4). Rotavirus infection often leads to diarrhea in suckling and weaned pigs that can be resolved in two or three days if not complicated by secondary bacterial infections (1). In contrast, when rotavirus infection occurs in combination with enteric bacteria, such as enterotoxigenic *Escherichia coli* (ETEC) or *Clostridium perfringens*, a higher severe disease can be developed conducting to dehydration and diarrhea endangering the life of the animal (5, 6). Then, rotavirus infections and rotavirus/bacteria superinfections are associated with a great economic impact in livestock industry due to increased mortality in young animals, the elevated cost of treatments and the diminished growth in animals that recover from the disease (1, 2).

The mechanisms involved in the collaboration of pathogenic bacteria and virus in promoting disease development has recently gained attention. The most severe effect of infections caused by viruses and bacteria acting together has been associated with two mechanisms that are not mutually exclusive. On the one hand, direct interactions occur when one pathogen exploits a component of the other to facilitate its penetration into the host cells (7, 8). On the other hand, indirect interactions result in increased tissue damage and alteration of the immune response as a consequence of the infection with one pathogen that facilitates the colonization and spreading of the second (9, 10). Then, alteration of epithelial barriers, cell loss, altered mucus secretion, unregulated inflammatory responses or immune suppression were described as mechanisms by which enteric pathogens can potentiate secondary infections in the gut. In this regard, rotavirus is recognized in the intestinal mucosa by germ-line-encoded pattern-recognition receptors (PRRs) including Toll-like receptor (TLR)-3, retinoic acid-inducible gene-I (RIG-I), and melanoma differentiation-associated gene-5 (MDA-5), stimulating cellular signaling cascades that culminate in the expression of type I interferons (IFNs), antiviral factors and inflammatory cytokines and chemokines that orchestrate the local innate immune response to react to viral infection (9, 10). Although this response is necessary to eliminate the virus, if it is not properly regulated it can be harmful to the host. In fact, it was shown that rotavirus is able to induce severe mucosal damage in the gut *via* TLR3-mediated inflammation including villous atrophy, mucosal erosion, and gut wall attenuation (11). Similarly, infection with Gram negative pathogens like ETEC can stimulate intestinal inflammatory responses *via* the activation of PRRs such as TLR4. Upon recognition of its cognate bacterial ligand, TLR4 dimerizes and initiates a signaling pathway that conduct to the activation of a pro-inflammatory response designed to eliminate the pathogen. However, the dysregulated activation of the TLR4-mediated inflammatory response can cause the synthesis of high and sustained levels of pro-inflammatory cytokines such as interleukin (IL)-8 and monocyte chemoattractant protein-1 (MCP-1) that induce the recruitment and activation of inflammatory cells that contribute to damage to the intestinal mucosa (12, 13). These investigations suggest that the efficient modulation of the immune responses induced by pathogens in the context of superinfections could help to reduce the severity of the infectious diseases.

Antimicrobial compounds have been widely used to prevent and control gastrointestinal infections in pigs despite the fact that they have no effect on infections caused by viruses and that their indiscriminate usage is increasing the spread of antimicrobial resistance among bacterial pathogens (14). Therefore, effective alternatives for the prevention or treatment of intestinal infections in pigs are being actively searched by scientists across the globe. In the last decades, great advances have been made in the characterization of the cellular and molecular mechanisms involved in the intestinal immune responses of pigs to bacterial and viral pathogens. In addition, the influence of the porcine intestinal microbiota on the generation and regulation of such responses is being better clarified (15, 16). This new molecular information has been helpful to develop new strategies to prevent bacterial and viral induced diarrhea in the porcine host. Among these alternatives, beneficial microorganisms with the ability to modulate the mucosal immune system, referred to as immunobiotics, have been shown to be an interesting tool for improving the health of pigs and their resistance to infections (17, 18). Immunobiotics were shown to be capable of modulating both innate and adaptive immune responses against pathogens increasing their clearance and diminishing inflammatory-mediated intestinal injury (17). Of note, most of the immunobiotic strains tested for the prevention of infections in pigs are human strains such as *Lactobacillus rhamnosus* GG, *Bifidobacterium lactis* Bb12, or *E. coli* Nissle 1917 (19–23), while porcine-specific immunobiotics have been lees explored in the context of infections (24, 25).

In a recent study, we demonstrated that the feeding of pigs with wakame (*Undaria pinnatifida*), a popular and economically important edible alga in Asian countries (26), was able to modify the gastrointestinal microbiota inducing a significant increase on the abundance of *Ligilactobacillus salivarius* (27), [Basonym: *Lactobacillus salivarius* (28)]. Moreover, considering the reports that indicate that wakame feeding was associated to a beneficial modulation of the pigs´ immune system (29); we hypothesized that the increase in lactobacilli would be associated to the immunomodulatory effect of wakame. Then, we constructed a library of *L. salivarius* strains from wakame-fed pigs and investigated their capacities to modulate IFN-β expression in response to TLR3 activation in porcine intestinal epithelial (PIE) cells. Our results demonstrated a strain dependent ability in the improvement of IFN-β in PIE cells after TLR3 activation (27). In this work, we aimed to further characterize the immunomodulatory activities of *L. salivarius* strains from wakame-fed pigs by evaluating their ability to modulate the innate immune responses in PIE cells triggered by TLR3 or TLR4 activations. Two strains with a remarkable immunomodulatory potential were selected, *L. salivarius* FFIG35 and FFIG58, and their capacities to differentially modulate TLR3-triggered innate immune response in PIE cells, as well as the resistance to rotavirus infection were evaluated in detail. Moreover, a model of ETEC/rotavirus superinfection in PIE cells was developed and the ability of FFIG35 and FFIG58 to protect porcine cells against a more severe disease was also studied. In addition, *in vivo* studies performed in mice models confirmed the ability of *L. salivarius* FFIG58 to beneficially modulate the intestinal innate immune response and protect against the ETEC infection, which was induced after the stimulation of mice with poly(I:C) to induce TLR3-mediated intestinal inflammatory damage.

## Materials and Methods

### *Ligilactobacillus salivarius* Strains

*L. salivarius* strains were isolated form the mucus membrane of the small intestine (jejunum, jejunum Peyer’s patches, ileum, and ileum Peyer’s patches) of wakame-fed pigs as described previously (27). The *L. salivarius* strains isolated from the intestinal tract of wakame-fed pigs were designated as FFIG. Lactobacilli strains were grown in Man–Rogosa–Sharpe (MRS) broth at 37°C. For the *in vitro* immunomodulatory assays, overnight cultures were harvested by centrifugation, washed three times with sterile phosphate-buffered saline (PBS), counted in a Petroff–Hausser counting chamber, and resuspended in DMEM until use.

### PIE Cells and TLRs Activation

The PIE cell line was originally established at Tohoku University from intestinal epithelia of an unsuckled neonatal pig as described previously (30, 31). DMEM medium supplemented with 10% fetal calf serum (FCS), penicillin (100 mg/mL), and streptomycin (100 U/mL) was used for the maintenance of PIE cells. The cells (3.0 × 10^4^ per well) were grown in 12 well type I collagen (bovine dermis) coated plates at 37°C in a humidified atmosphere of 5% CO_2_. After 3 days of culturing period, 1 mL of DMEM containing the different *L. salivarius* strains isolated from the intestine of wakame-fed pigs (5 × 10^7^ cells/mL) were added to PIE cells monolayers. Lactobacilli stimulation was performed from 3 to 48 hours. The expressions of several immune factors were determined by RT-qPCR as described below.

In a second set of experiments, PIE cells were stimulated with *L. salivarius* strains (5 × 10^7^ cells/mL) for 48 hours at 37°C, 5% CO_2_. PIE cells were washed with fresh medium to eliminate lactobacilli and subsequently stimulated with 10 ug/mL of poly(I:C) (Sigma Aldrich, USA) or enterotoxigenic *Escherichia coli* (ETEC) (5 × 10^6^ cells/mL) to induce the activation of TLR3 and TLR4, respectively. Stimulation with poly(I:C) or ETEC were performed from 3 to 12 hours. The expressions of several immune factors were determined by RT-qPCR as described below. ETEC strain O9:H-, F6 pilus +, heat-stable enterotoxin (STa) + and was kindly provided by Dr. Nakazawa at the National Institute of Animal Health (Tsukuba, Japan).

### Rotavirus

A rotavirus strain isolated from pigs (OSU) was used in this study. Obtention of rotavirus for infection experiments was performed as described previously (32). Briefly, rotavirus OSU was treated with 10 μg/mL trypsin (Sigma, Type I) at 37°C for 30 minutes and inoculated onto confluent MA104 cells. After an hour of absorption, the inoculum was removed, and the cells were incubated with serum-free MEM containing 1 μg/mL trypsin at 37°C. When the cytopathic effect reached more than 80%, the culture supernatant was harvested by three rounds of freezing and thawing process. The virus stock was stored at -80°C for further experiments.

### PIE Cells and Rotavirus Infection

PIE cells were plated at 3.0 × 10^4^ cells/well in 12 well type I collagen coated plates (SUMILON, Tokyo, Japan) and incubated at 37°C, 5% CO_2_. After 8 days of culturing, cells were pre-stimulated with *L. salivarius* strains isolated from the intestine of wakame-fed pigs (5 × 10^7^ cells/mL). Then, cells were washed three times with DMEM medium to eliminate the bacteria and subsequently inoculated with trypsin-activated rotavirus OSU at multiplicity of infection (MOI) 1. At hour 16 post-inoculation, PIE cells were fixed after removal of the inoculums and the infected virus titer were analyzed by immunofluorescence staining.

The immunofluorescence staining for detection of cells infected with rotavirus was performed as described previously (32). Briefly, rotavirus challenged-PIE cells were fixed with 80% acetone at 4°C for 15 minutes. Then, cells were washed twice with PBS, and subsequently incubated with a guinea pig anti-rotavirus Wa strain polyclonal antibody (1:750 in PBS, 50 μL/well) for 30 minutes at 37°C. Following three washes with PBS, cells were incubated at 37°C for 30 minutes with Fluorescein isothiocyanate (FITC) conjugated anti-guinea pig IgG (H+L) antibody (Rockland antibodies and assays, Limerick, PA, 1:400 in PBS, 50 μL/well). Infected cells were examined and photographed under an immunofluorescence microscope (Confocal laser microscope, MRC-1024, Bio-Rad, Richmond, CA) after three rounds of washing with PBS and mounted with 30% glycerol prepared in PBS. The number of infected cells in the control was set to 100% and the number of infected cells in the lactobacilli-stimulated group was used to calculate the percentage. In addition, the expressions of several immune factors were determined by RT-qPCR as described below, after 3 to 12 hours of rotavirus infection.

In a second set of experiments, PIE cells were superinfected with ETEC and rotavirus. Cultured PIE cells were stimulated with ETEC for 12 hours and then challenged with rotavirus as described above. Rotavirus titers were determined after 16 hours by immunofluorescence staining while the expressions of immune factors were assessed by RT-qPCR after 3 to 12 hours of rotavirus infection.

### RT-qPCR

The expression of immune factors in PIE cells were studied as described previously (30, 31). Briefly, total RNA was extracted with TRIzol reagent (Invitrogen) and its purity and quantity were analyzed by Nano drop spectrophotometer ND-1000 UV-Vis (NanoDrop Technologies, USA). The RNA (500 ng) was used to synthesize cDNA by Thermal cycler (BIO-RAD, USA) with the Quantitect reverse transcription (RT) kit (Qiagen, Tokyo, Japan) following the manufacturer instructions. The qPCR was performed in a 7300 real-time PCR system (Applied Biosystems, Warrington, UK) with platinum SYBR green (qPCR supermix uracil-DNA glycosylase with 6- carboxyl-X-rhodamine, Invitrogen). The primers for the analysis of immune factors were described before (31, 32) or are listed in **Supplementary Table 1**. For the PCR reaction 2.5 μL of cDNA were mixture with 7.5 μL of master mix that included RT enzyme, SYBR green, forward and reverse primers (1 pmol/μL). The reaction cycles were performed as follow: 50°C for 5min; 95°C for 5min; 40 cycles at 95°C for 15 s, 60°C for 30 s and finally 72°C for 30 s. According to the minimum information for publication of quantitative real-time PCR experiments guidelines, β-actin was used as a housekeeping gene because of its high stability across porcine various tissues (33, 34), including PIE cells in the context of viral (30, 31) and bacterial (35–37) infections. The expression of the housekeeping gene was used to normalize cDNA levels for differences in total cDNA levels in the samples. A relative index was calculated after normalization with β-actin and results were expressed as normalized fold expression based on cell controls set as 1.0.

### Mice and Ethical Statement

Female 5-week-old BALB/c mice were obtained from the closed colony kept at CERELA-CONICET (Tucuman, Argentina). Animals were housed in plastic cages in a controlled atmosphere (22 ± 2°C temperature, 55 ± 2% humidity) with a 12 h light/dark cycle.

Mice were housed in plastic cages and environmental conditions were kept constant, in agreement with the standards for animal housing. Animal welfare was in charge of researchers and special staff trained in animal care and handling at CERELA. The minimal number of mice required for an appropriate statistical analysis was calculated with the help of the Biostatistics Laboratory of CERELA. Mice health and behavior were monitored twice a day. Animals were euthanized immediately after the time point was reached by using xylazine and ketamine. No signs of discomfort or pain were observed before mice reached the endpoints. No deaths were observed before mice reached the endpoints.

All experiments were carried out in compliance with the Guide for Care and Use of Laboratory Animals and approved by the Ethical Committee of Animal Care at CERELA, Argentina (protocol numbers BIOT-CRL/14 and BIOT-CRL/11).

### Poly(I:C) and Poly(I:C)/ETEC Challenge in Mice

*L. salivarius* FFIG58 was orally administered to different groups of mice for 5 consecutive days at a dose of 10^8^ cells/mouse/day. The immunobiotic strain *Lacticaseibacillus rhamnosus* CRL1505 was used for comparisons. The lactobacilli-treated groups and the untreated control mice were fed a conventional balanced diet *ad libitum*. One day after the las lactobacilli administration (day 6) animals were challenged as described below. Two set of experiment were performed in lactobacilli-treated and control mice. In the first set of experiments, mice were challenged by the intraperitoneal route with 100 μL of PBS containing 30 μg poly(I:C) to induce the activation of TLR3 in the intestinal mucosa according to our previous publications (38, 39). In the second set of experiments, mice were intraperitoneally challenged with poly(I:C) and two days after they were orally inoculated with 200 μL of a bacterial suspension containing the human ETEC O9, F4 pilus +, STp + kanamycin resistant strain (1 × 10^9^ cells) diluted with 0.1 M carbonate buffer (pH 9.0). Two days after the ETEC inoculation, the mice were sacrificed to collect the jejunum, ileum, spleen, and liver samples. The collected tissues were weighed and homogenized in BHI broth. Homogenates were plated on kanamycin containing MAC agar plates for ETEC counts (40). Results were expressed as log of colony-forming units (CFU) per gram of organ.

Serum biochemical markers of injury as well as intestinal cytokines ´concentrations were evaluated two days after poly(I:C) administration or two days after ETEC challenge as described below.

### Markers of Injury and Cytokine Concentrations

Lactate dehydrogenase (LDH) and aspartate aminotransferase (AST) activities were determined in the serum to evaluate gastrointestinal injury indirectly. Blood samples were obtained through cardiac puncture under anesthesia. LDH and AST activities, expressed as units per liter of serum, were determined by measuring the formation of the reduced form of nicotinamide adenine dinucleotide (NAD) using the Wiener reagents and procedures (Wiener Lab, Buenos Aires, Argentina) (38, 39).

Intestinal fluid samples were obtained as described before (38, 39). Briefly, the small intestine was flushed with 5 ml of PBS and the fluid was centrifuged (10,000 g, 4°C 10 min) to separate particulate material. The intestinal supernatant samples were kept frozen at -80°C until use. Tumor necrosis factor (TNF)-α, IL-6, IL-10, IL-15, interferon (IFN)-β and IFN-γ, chemokine KC (or CXCL1), and MCP-1 concentrations in intestinal fluid a were measured with commercially available enzyme-linked immunosorbent assay (ELISA) technique kits following the manufacturer’s recommendations (R&D Systems, MN, USA).

### Statistical Analysis

Statistical analyses were performed using the GLM and REG procedures available in the SAS computer program (SAS, 1994). Comparisons between mean values were carried out using one-way analysis of variance and Fisher’s least-significant-difference (LSD) test. For these analyses, P values of < 0.05 were considered significant.

## Results

### Screening of Porcine *L. salivarius* Strains With the Ability to Modulate Innate Immune Responses in PIE Cells

**Figure 1:**
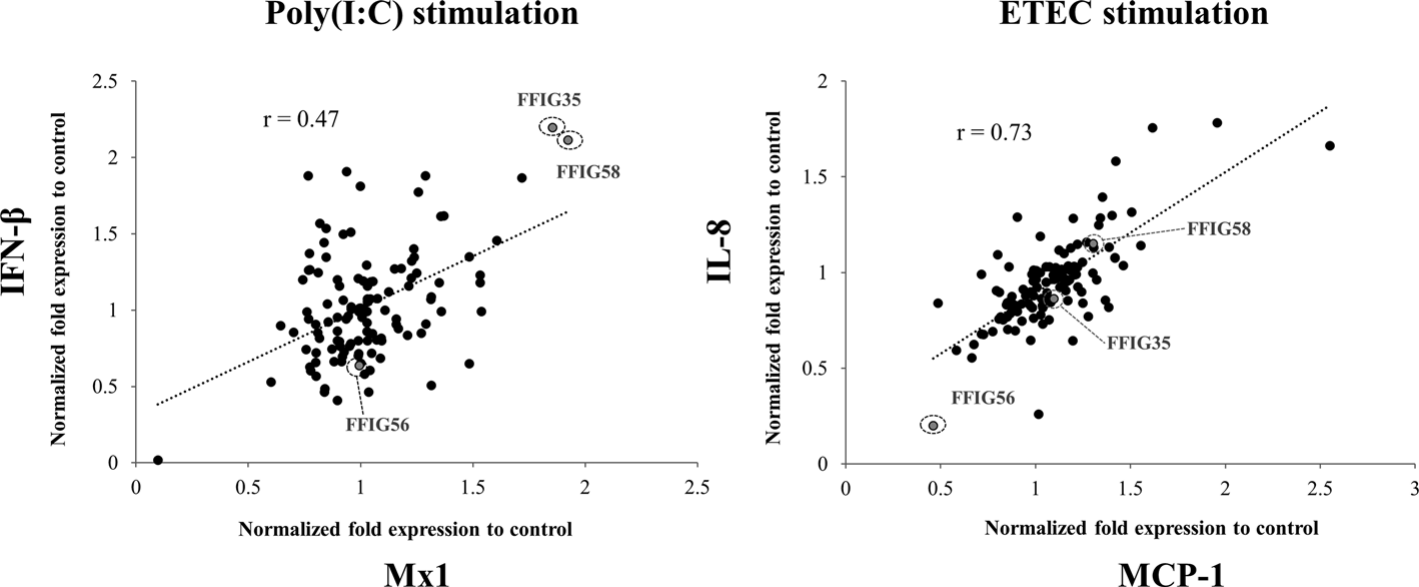
Effect of porcine *Ligilactobacillus salivarius* strains on the innate immune responses of porcine intestinal epithelial (PIE) cells triggered by the activation of Toll-like receptor 3 (TLR3) or TLR4. PIE cells were stimulated with different *L. salivarius* strains isolated form the porcine gastrointestinal tract and challenged with poly(I:C) or enterotoxigenic *Escherichia coli* (ETEC) to induce the activation of TLR3 and TLR4, respectively. The expressions of interferon (IFN)-β, and the antiviral factor Mx1 were analyzed by RT-qPCR after 12 hours of TLR3 activation. The expression of interleukin (IL)-8 and monocyte chemoattractant protein 1 (MCP-1) were analyzed by RT-qPCR after 12 hours of TLR4 activation. PIE cells stimulated only with poly(I:C) or ETEC F6 were used as controls. Results are expressed as normalized fold expression to control (lactobacilli treated vs. non-lactobacilli-treated cells). The correlation between the expressions of immune genes was assessed by a linear regression function and coefficient of determination. Experiments were performed in triplicate. Only one set of data is shown in the figure for clarity.

Previously, we isolated several *L. salivarius* strains from the gut of wakame-fed pigs and preliminary studies confirmed a strain-dependent capacity in their ability to modulate IFN-β expression in PIE cells in response to TLR3 activation (27). In this work, we aimed to further select the strains with the highest immunomodulatory potential. For this purpose, porcine *L. salivarius* strains were used to stimulate PIE cells, which were then challenged with poly(I:C) to trigger TLR3-mediated inflammation, and the expression of *IFN-β* and *Mx1* was evaluated (**Figure 1**). In a second set of experiments, PIE cells were stimulated with porcine *L. salivarius* strains and then challenged with ETEC to induce the activation of TLR4. The expression of *IL-8* and *MCP-1* in response to ETEC were determined for each strain (**Figure 1**) considering that both cytokines were described to participate in the intestinal epithelial damage induced by TLR4-mediated inflammation (12, 13). The analysis of the correlation between each immune factor within the poly(I:C) or ETEC challenges groups by a linear regression function and coefficient of determination confirmed a strain-dependent immunomodulatory potential. Among the strains evaluated, *L. salivarius* FFIG35 and FFIG58 stood out for their ability to improve the expression of *IFN-β* and *Mx1* in poly(I:C)-challenged PIE cells, indicating their antiviral potential. On the other hand, *L. salivarius* FFIG56 was the strain with the highest ability to reduce the expression of *IL-8* and *MCP-1* in ETEC-challenged cells (**Figure 1**). Then, *L. salivarius* FFIG35, FFIG56, and FFIG58 were selected for further experiments.

### Effect of Porcine *L. salivarius* Strains on the Expression of Immune Factors in PIE Cells

**Figure 2:**
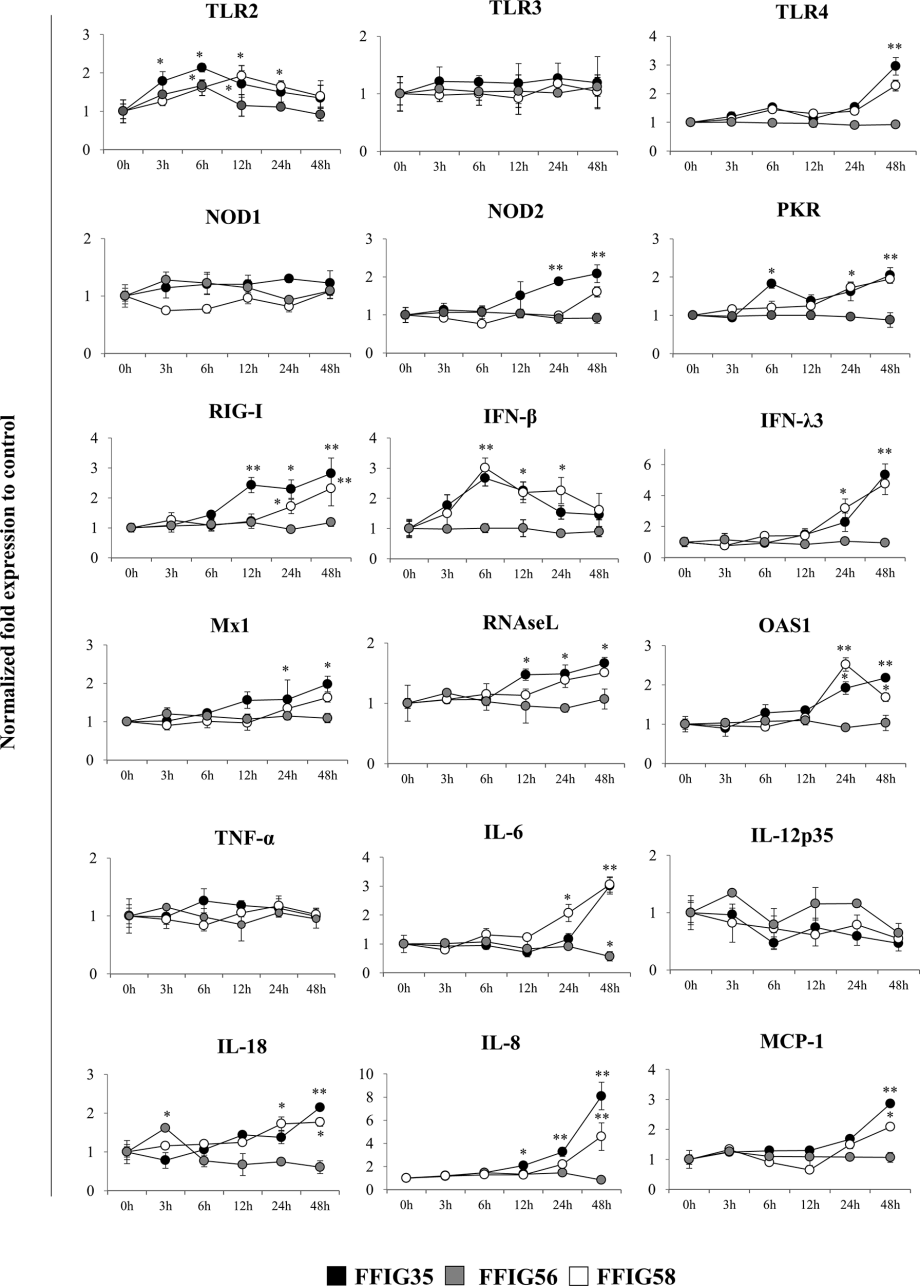
Effect of porcine *Ligilactobacillus salivarius* strains on the expression of immune factors in porcine intestinal epithelial (PIE) cells. PIE cells were stimulated with *L. salivarius* FFIG35, FFIG56 or FFIG58 isolated form the porcine gastrointestinal tract and the expression of Toll-like receptor (TLR)-2, TLR3, TLR4, nucleotide-binding oligomerization domain-containing protein (NOD)-1, NOD2, protein kinase R (PKR), retinoic acid-inducible gene I (RIG-I), interferon (IFN)-β, IFN-λ3, IFN-induced GTP-binding protein Mx1 (Mx1), ribonuclease L (RNAseL), 2’-5’-oligoadenylate synthetase 1 (OAS1), tumor necrosis factor (TNF)-α, interleukin (IL)-6, IL-8, IL-12, IL-18 and monocyte chemoattractant protein 1 (MCP-1) were determined by RT-qPCR at the indicated time points. After normalization of genes with β-actin, the relative expression compared to the expression of each gene in the control was calculated. The results represent data from three independent experiments at each time point. Values are means ± SD. Asterisks indicate significant differences when compared to hour 0 (*P < 0.05, **P < 0.01).

We next aimed to evaluate whether the selected *L. salivarius* strains were able to differentially modulate the expression of several immune factors in the absence of inflammatory stimuli. Then, PIE cells were stimulated with FFIG35, FFIG56, or FFIG58 strains and the expression of pattern recognition receptors (PRRs), IFNs and antiviral factors as well as inflammatory cytokines and chemokines were determined at different time points (**Figure 2**). The three strains increased the expression of *TLR2* in PIE cells although with different kinetics. An earlier increase of *TLR2* with a peak at hour 6 was observed for *L. salivarius* FFIG35. This peak was also observed for the FFIG56 although the values were lower than the observed in FFIG35-treated PIE cells. *TLR2* expression had a peak at hour 12 in PIE cells stimulated with *L. salivarius* FFIG58. The expression of *TLR4* was modulated by FFIG35 and FFIG58 at hour 48 and a significant increase for this PRR was observed in the mentioned groups when compared to the basal expression or FFIG56-treated PIE cells (**Figure 2**). None of the strains induced changes in the expression of *TLR3* or *NOD1*. In addition, *L. salivarius* FFIG56 was not capable of modulating the expressions of NOD2, protein kinase RNA-activated (*PKR*) or *RIG-I* in any of the time points evaluated. On the contrary, both FFIG35 and FFIG58 were able to significantly increase the expressions of *NOD2, PKR* or *RIG-I*, especially after 24 hours of stimulation. *L. salivarius* FFIG35 was more efficient than the FFIG58 strain to up-regulate *NOD2* and *RIG-I*. Both FFIG35 and FFIG58 showed a remarkable ability to increase the expression *IFN-β* and *IFN-λ3* (**Figure 2**). While *IFN-β* was increased between hours 6 and 24, *IFN-λ3* was up-regulated after hour 24 in FFIG35- and FFIG58-treated PIE cells. Consistent with the improved IFNs expression, enhanced levels of *Mx1, RNAseL* and *OAS1* were found in PIE cells treated with *L. salivarius* FFIG35 or FFIG58 between hours 12 and 48. Of note, *L. salivarius* FFIG56 was not able to induce modifications in the expressions of *IFN-β, IFN-λ3, Mx1, RNAseL* or *OAS1*. When inflammatory cytokines and chemokines were evaluated, it was observed that none of the strains induced changes in the expression of *TNF-α* or *IL-12p35* in PIE cells (**Figure 2**). *L. salivarius* FFIG58 and FFIG35 significantly increased *IL-6* expression from hours 24 and 48, respectively, while FFIG56 decreased this cytokine at hour 48. In addition, FFIG35 and FFIG58 up-regulated the expressions of *IL-8* and *MCP-1* being the later strain the most efficient to induce this effect. No modification in the expressions of *IL-8* or *MCP-1* were found in PIE cells treated with *L. salivarius* FFIG56. An increase in the expression of *IL-18* was found at hour 3 for PIE cells stimulated with the FFIG56 strain, while *L. salivarius* FFIG58 and FFIG35 significantly increased this cytokine from hours 24 and 48, respectively (**Figure 2**).

### Effect of Porcine *L. salivarius* Strains on TLR3-Triggered Innate Immune Response in PIE Cells

**Figure 3:**
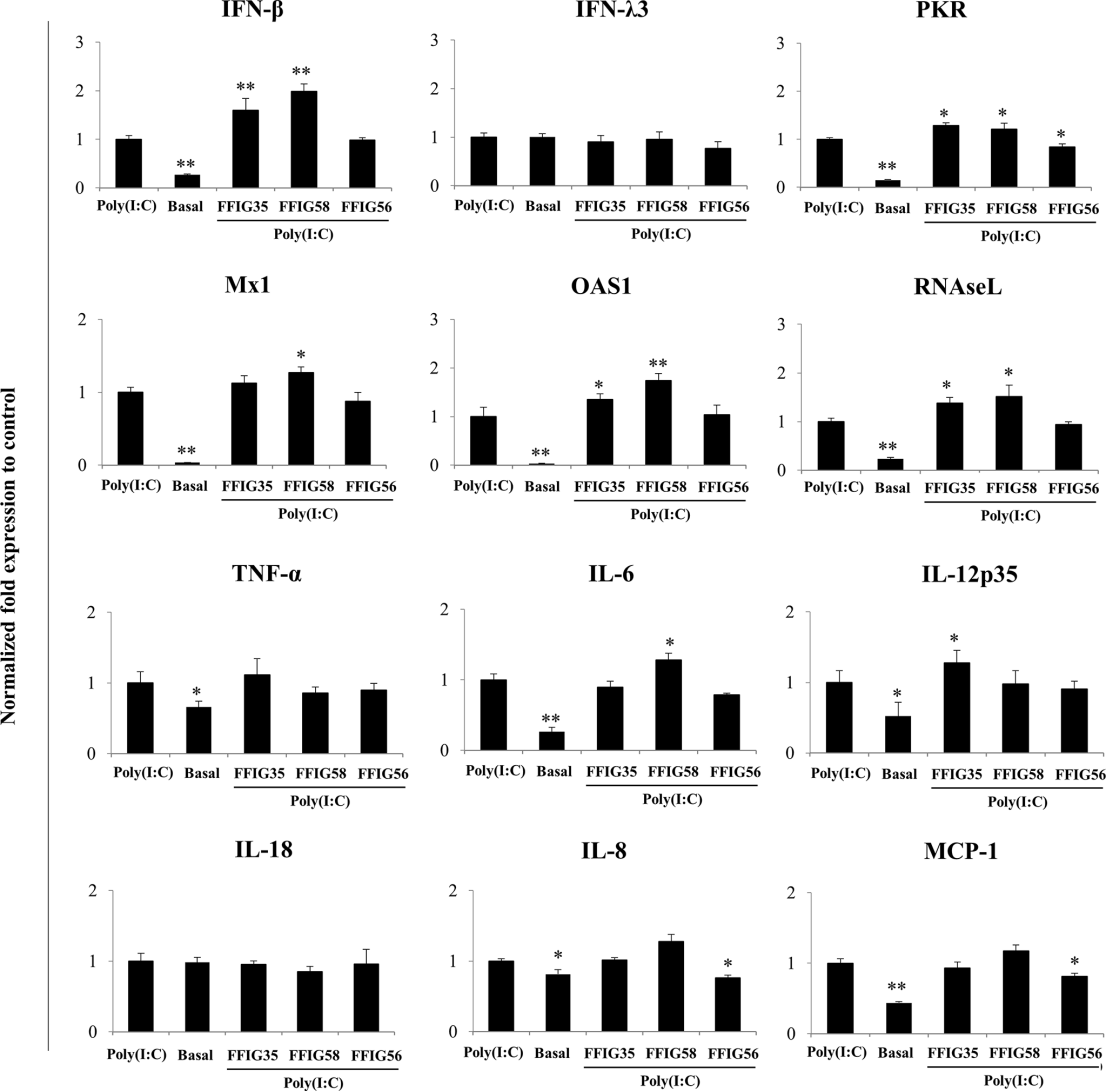
Effect of porcine *Ligilactobacillus salivarius* strains on the expression of immune factors in porcine intestinal epithelial (PIE) cells in response to Toll-like receptor (TLR)-3 activation. PIE cells were stimulated with *L. salivarius* FFIG35, FFIG56 or FFIG58 isolated form the porcine gastrointestinal tract and then challenged with poly(I:C) to activate TLR3. The expression of interferon (IFN)-β, IFN-λ3, protein kinase R (PKR), IFN-induced GTP-binding protein Mx1 (Mx1), ribonuclease L (RNAseL), 2’-5’-oligoadenylate synthetase 1 (OAS1), tumor necrosis factor (TNF)-α, interleukin (IL)-6, IL-8, IL-12, IL-18 and monocyte chemoattractant protein 1 (MCP-1) were determined by RT-qPCR after 12 hours of TLR3 activation. PIE cells with no challenge (basal control) or stimulated only with poly(I:C) (poly(I:C) control) were used for comparisons. After normalization of genes with β-actin, the relative expression compared to the expression of each gene in the poly(I:C) control was calculated. The results represent data from three independent experiments. Values are means ± SD. Asterisks indicate significant differences when compared to the poly(I:C) control group (*P < 0.05, **P < 0.01).

A more detailed study of the influence of the selected strains on the PIE cells response to poly(I:C) was then performed. As shown in **Figure 3**, we confirmed the ability of FFIG35 and FFIG58 to significantly up-regulate the expression of *IFN-β* after TLR3 activation while *L. salivarius* FFIG56 was not capable of achieving this effect. Although FFIG35 and FFIG58 were able to increase the expression of *IFN-λ3* in non-inflammatory conditions, no effect was observed after poly(I:C) stimulation. In addition, no effect on *IFN-λ3* expression was detected in PIE cells stimulated with *L. salivarius* FFIG56. A significant increase in the expressions of *RNAseL* and *OAS1* was observed in FFIG35- and FFIG58-treated cells, while only *L. salivarius* FFIG58 increased *Mx1* expression. On the contrary, no effect on the antiviral factors expressions was observed for the FFIG56 strain. Of note, the three lactobacilli increased *PKR* expression in poly(I:C)-challenged PIE cells (**Figure 3**).

No differences in the expressions of *TNF-α* or *IL-18* were observed in *L. salivarius*-treated PIE cells when compared to control cells after poly(I:C) challenge (**Figure 3**). *L. salivarius* FFIG58 and FFIG35 increased the expressions of *IL-6* and *IL-12p35*, respectively. Of note, the FFIG58 strain significantly reduced the expressions of *IL-8* and *MCP-1* in poly(I:C)-challenged PIE cells (**Figure 3**).

**Figure 4:**
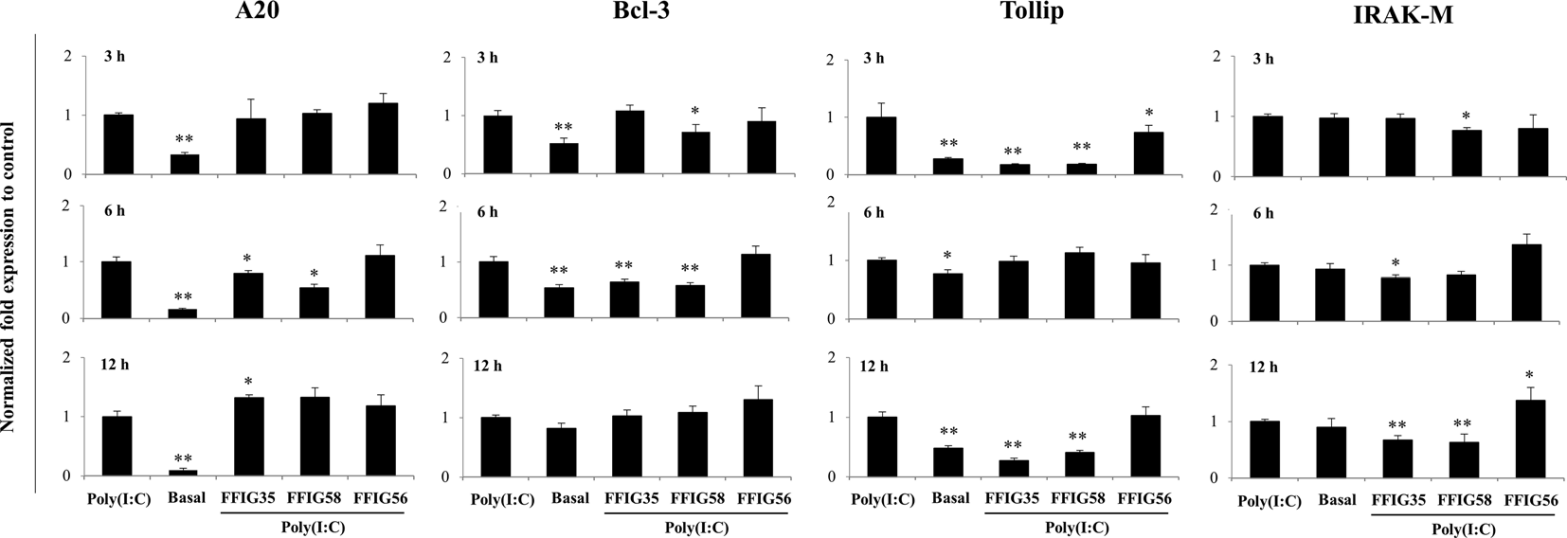
Effect of porcine *Ligilactobacillus salivarius* strains on the expression of negative regulators of the Toll-like receptor (TLR) signaling pathway in porcine intestinal epithelial (PIE) cells in response to TLR3 activation. PIE cells were stimulated with *L. salivarius* FFIG35, FFIG56 or FFIG58 isolated form the porcine gastrointestinal tract and then challenged with poly(I:C) to activate TLR3. The expression of zinc finger protein A20 (A20), B-cell lymphoma-3 (Bcl-3), Toll interacting protein (Tollip) and interleukin-1 receptor-associated kinase M (IRAK-M) were determined by RT-qPCR after 3, 6 or 12 hours of TLR3 activation. PIE cells with no challenge (basal control) or stimulated only with poly(I:C) (poly(I:C) control) were used for comparisons. After normalization of genes with β-actin, the relative expression compared to the expression of each gene in the poly(I:C) control was calculated. The results represent data from three independent experiments. Values are means ± SD. Asterisks indicate significant differences when compared to the poly(I:C) control group (*P < 0.05, **P < 0.01).

Our previous works indicated that the ability of immunomodulatory lactobacilli to differentially regulate the expression of type I IFNs, antiviral factors and cytokines in PIE cells in response to TLR3 activation are related to their capacity to modulate the expression of negative regulators of the TLR signaling pathway (30, 32). Then, we next evaluated the influence of the *L. salivarius* strains on the expressions of *A20, Bcl-3, Tollip, IRAK-M* (**Figure 4**), *MKP-1* and *SIGIRR* (**Supplementary Figure 1**). As shown in **Figure 4**, *L. salivarius* FFIG58 and FFIG35 reduced the expression of *A20* at hour 6 post-poly(I:C) stimulation. Both, FFIG35 and FFIG58 diminished the expression of *Bcl-3* (hour 6) and *Tollip* (hours 3 and 12). In addition, a slightly but significant reduction of *Tollip* expression was found at hour 3 post-poly(I:C) stimulation in PIE cells treated with *L. salivarius* FFIG56 (**Figure 4**). *L. salivarius* FFIG58 and FFIG35 reduced the expression of *IRAK-M* at hours 3 and 6, respectively while both strains reduced this factor at hour 12. *L. salivarius* FFIG35 diminished the expressions of *MKP-1* and *SIGIRR* at hour 6 while no effect was observed when these factors were evaluated in FFIG58-treated PIE cells (**Supplementary Figure 1**). Of note, the FFIG56 strain up-regulated the expression of *IRAK-M* at hour 12 post-poly(I:C) stimulation (**Figure 4**). Moreover, this was the only *L. salivarius* strain capable of increasing the expression of *MKP-1* at all the time points evaluated (**Supplementary Figure 1**).

### Effect of Porcine *L. salivarius* Strains on Rotavirus-Triggered Innate Immune Response in PIE Cells

**Figure 5:**
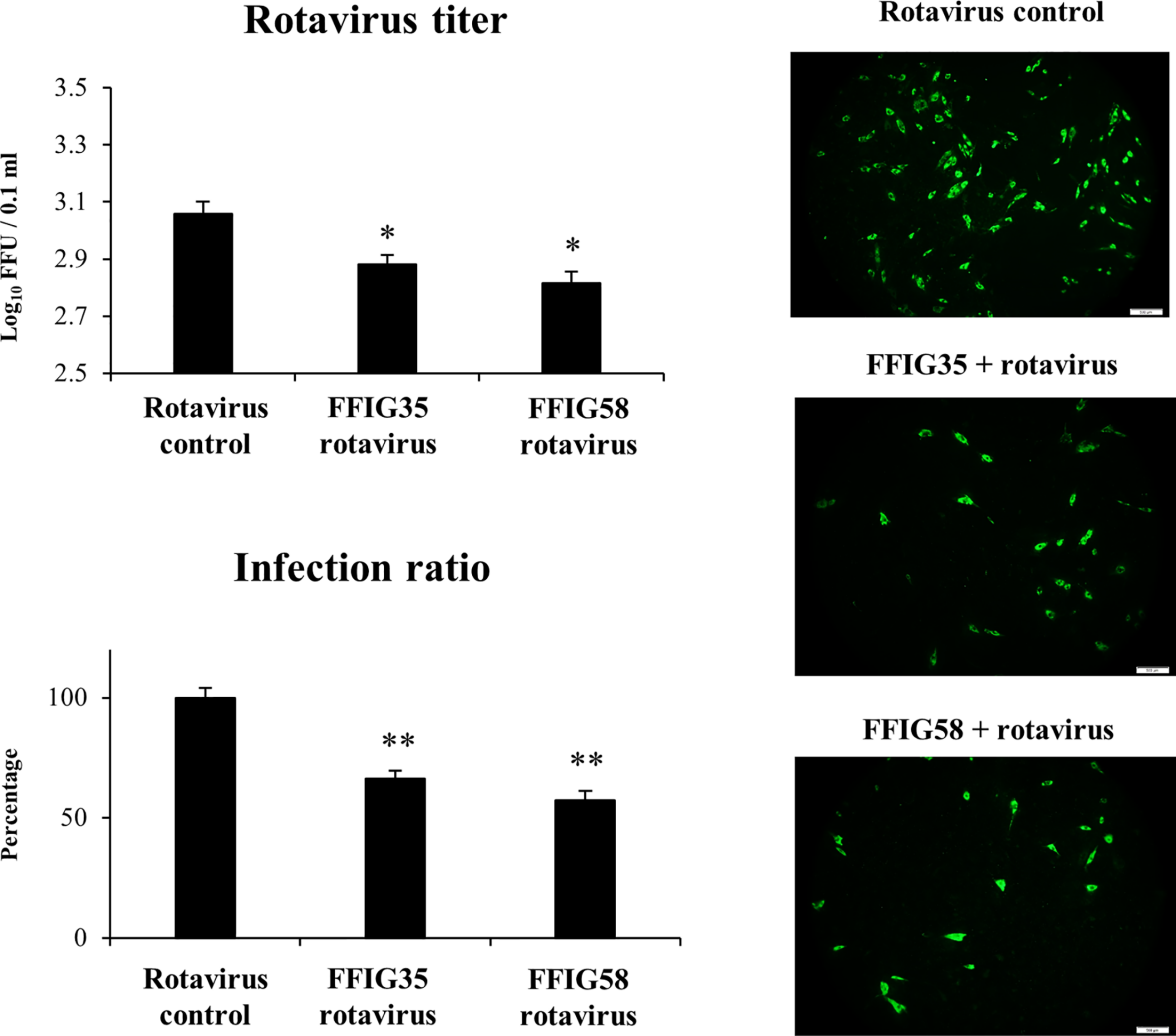
Effect of porcine *Ligilactobacillus salivarius* strains on the resistance of porcine intestinal epithelial (PIE) cells to rotavirus infection. PIE cells were stimulated with *L. salivarius* FFIG35 or FFIG58 isolated form the porcine gastrointestinal tract and then challenged with rotavirus. PIE cells with no lactobacilli treatment and challenged with rotavirus were used for comparisons. Rotavirus infection was evaluated by immunofluorescence assay. The cells with specific green fluorescence in the cytoplasm were photographed by confocal laser microscopy after labeling with fluorescence anti-rotavirus antibody. The protective ability of lactobacilli was examined by calculating the virus titer and the infection ratio. The results represent data from three independent experiments. Values are means ± SD. Asterisks indicate significant differences when compared to the rotavirus control group (*P < 0.05, **P < 0.01).

**Figure 6:**
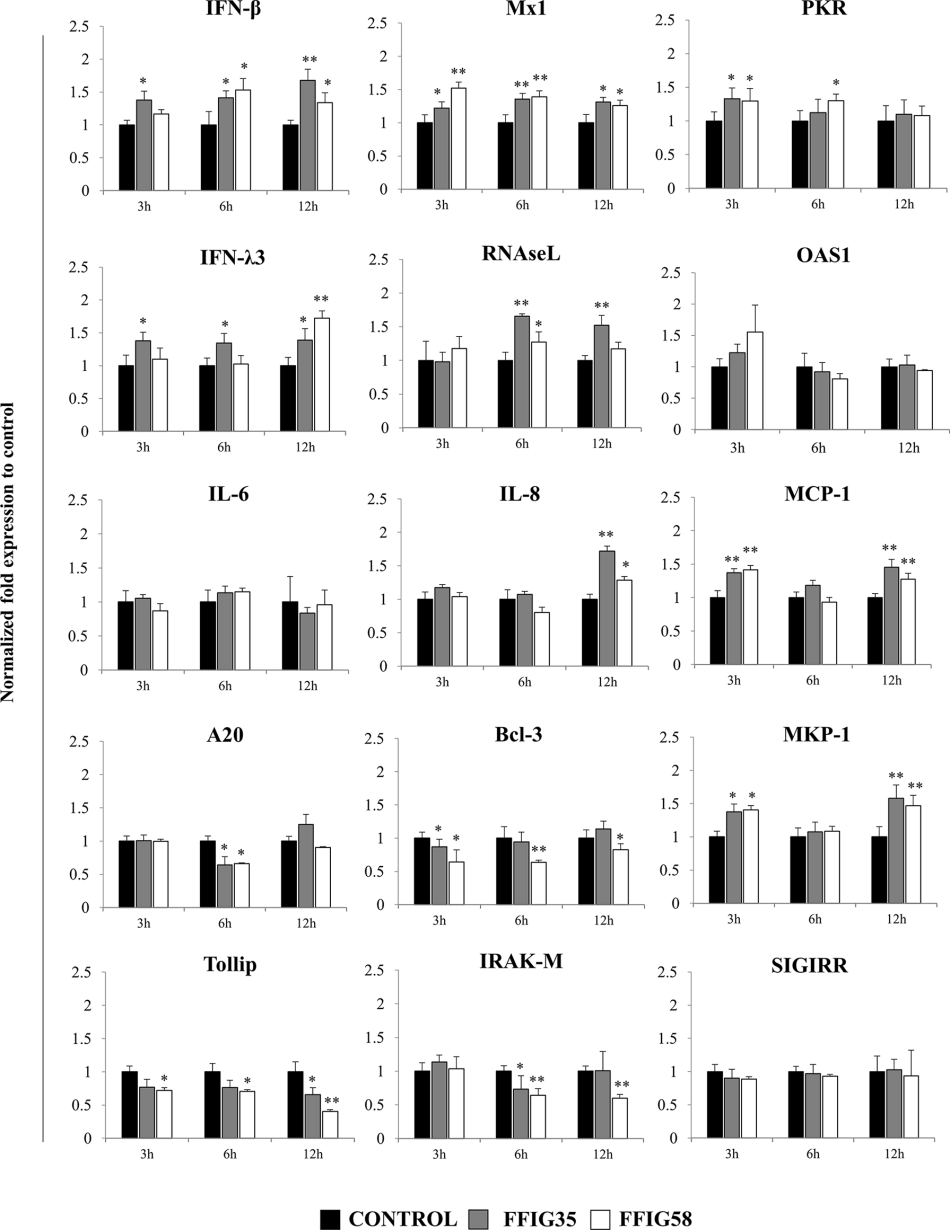
Effect of porcine *Ligilactobacillus salivarius* strains on the expression of immune factors in porcine intestinal epithelial (PIE) cells in response to rotavirus infection. PIE cells were stimulated with *L. salivarius* FFIG35 or FFIG58 isolated form the porcine gastrointestinal tract and then challenged with rotavirus. The expression of interferon (IFN)-β, IFN-λ3, protein kinase R (PKR), IFN-induced GTP-binding protein Mx1 (Mx1), ribonuclease L (RNAseL), 2’-5’-oligoadenylate synthetase 1 (OAS1), interleukin (IL)-6, IL-8, monocyte chemoattractant protein 1 (MCP-1), zinc finger protein A20 (A20), B-cell lymphoma-3 (Bcl-3), Toll interacting protein (Tollip), interleukin-1 receptor-associated kinase M (IRAK-M), mitogen-activated protein kinase phosphatase-1 (MKP-1) and single immunoglobulin interleukin-1 related receptor (SIGIRR) were determined by RT-qPCR after 3, 6 or 12 hours of rotavirus infection. PIE cells with no lactobacilli treatment and challenged with rotavirus were used for comparisons. After normalization of genes with β-actin, the relative expression compared to the expression of each gene in the rotavirus control was calculated. The results represent data from three independent experiments at each time point. Values are means ± SD. Asterisks indicate significant differences when compared to the rotavirus control group (*P < 0.05, **P < 0.01).

Taking into consideration that *L. salivarius* FFIG58 and FFIG35 had a remarkable ability to modulate the innate immune response triggered by TLR3 in PIE cells, we aimed to evaluate whether these strains conferred protection against rotavirus infection. Then, PIE cells were treated with FFIG35 or FFIG58 and subsequently challenged with rotavirus (**Figure 5**). As we reported previously (32), PIE cells are susceptible to rotavirus infection. Interestingly, both FFIG35 and FFIG58 were capable of significantly reducing the rotavirus titers as well as infection ratio in challenged PIE cells. Moreover, both strains were equally effective for inducing the protective effect against viral infection. We also evaluated the influence of lactobacilli treatments on the rotavirus-induced immune response in PIE cells. As shown in **Supplementary Figure 2**, rotavirus infection increased the expression of *IFN-β* and *IFN-λ3* as well as the antiviral factors *PKR, Mx1*, and *RNAseL* in PIE cells. Of note, *OAS1* was not modified by rotavirus infection in any of the time points evaluated. PIE cells treated with FFIG35 and FFIG58 strains before rotavirus infection had significantly higher levels of *IFN-β* and *IFN-λ3* than control cells (**Figure 6**). None of the strains induced significant differences in the expression of *OAS1* after rotavirus infection, while both *L. salivarius* strains increased the expressions of *PKR, Mx1* and *RNAseL*. *L. salivarius* FFIG35 was more efficient for increasing *RNAseL* while the FFIG58 strain was more efficient for the up-regulation of *PKR* (**Figure 6**).

Rotavirus infection also enhanced the expression of *IL-6, IL-8*, and *MCP-1* in PIE cells (**Supplementary Figure 3**). In addition, we observed that the treatment of PIE cells with *L. salivarius* FFIG35 or FFIG8 did not influenced the expression of *IL-6* (**Figure 6**). On the contrary, both lactobacilli strains increased the expressions of *IL-8* (hour 12) and *MCP-1* (hours 3 and 12) when compared to control PIE cells (**Figure 6**).

When the negative regulators of the TLR signaling pathway were investigated after infection of PIE cells with rotavirus, it was observed that both FFIG35 and FFIG58 were capable of reducing the expressions of *A20, Bcl-3, Tollip* and *IRAK-M* (**Figure 6**). *L. salivarius* FFIG58 significantly reduced *Bcl-3* and *Tollip* in all the time points evaluated while the FFIG35 strain reduced those regulators at hours 3 and 12, respectively. No changes in the expression of *SIGIRR* were observed in PIE cells treated with lactobacilli. In addition, a significant up-regulation of *MKP-1* at hours 3 and 12 was observed for PIE cells treated with *L. salivarius* FFIG58 or FFIG35 (**Figure 6**).

### Effect of Porcine *L. salivarius* Strains on ETEC-Triggered Innate Immune Response in PIE Cells

We also evaluated whether *L. salivarius* FFIG58 or FFIG35 modulated the inflammatory response induced in PIE cells by ETEC challenge. The stimulation of PIE cells with ETEC increased the expressions of *IFN-λ3* while the levels of *IFN-β, OAS1, PKR, Mx1*, and *RNAseL* were not modified when compared to basal levels (**Supplementary Figure 2**). No effect on the expression of *IFN-β* or antiviral factors was observed when PIE cells treated with FFIG35 or FFIG58 were analyzed (**Supplementary Figure 4**). A slight but significant decrease in the expression of *IFN-λ3* was observed at hour 12 post-ETEC challenge in FFIG58-treated PIE cells when compared to controls (**Supplementary Figure 4**). ETEC challenge enhanced the expressions of *IL-6, IL-8*, and *MCP-1* (**Supplementary Figure 2**). No effect on the expression of these inflammatory cytokines were observed for the FFIG35 treatment while *L. salivarius* FFIG58 induced a slight but significant decrease in the expression of *IL-6* and *IL-8* (**Supplementary Figure 5**). As expected, no differences between control PIE cells and cells treated with lactobacilli were found when the negative regulators of the TLR signaling pathways were evaluated after ETEC challenge (**Supplementary Figure 6**).

### Effect of Porcine *L. salivarius* Strains on ETEC-Rotavirus Superinfection in PIE Cells

**Figure 7:**
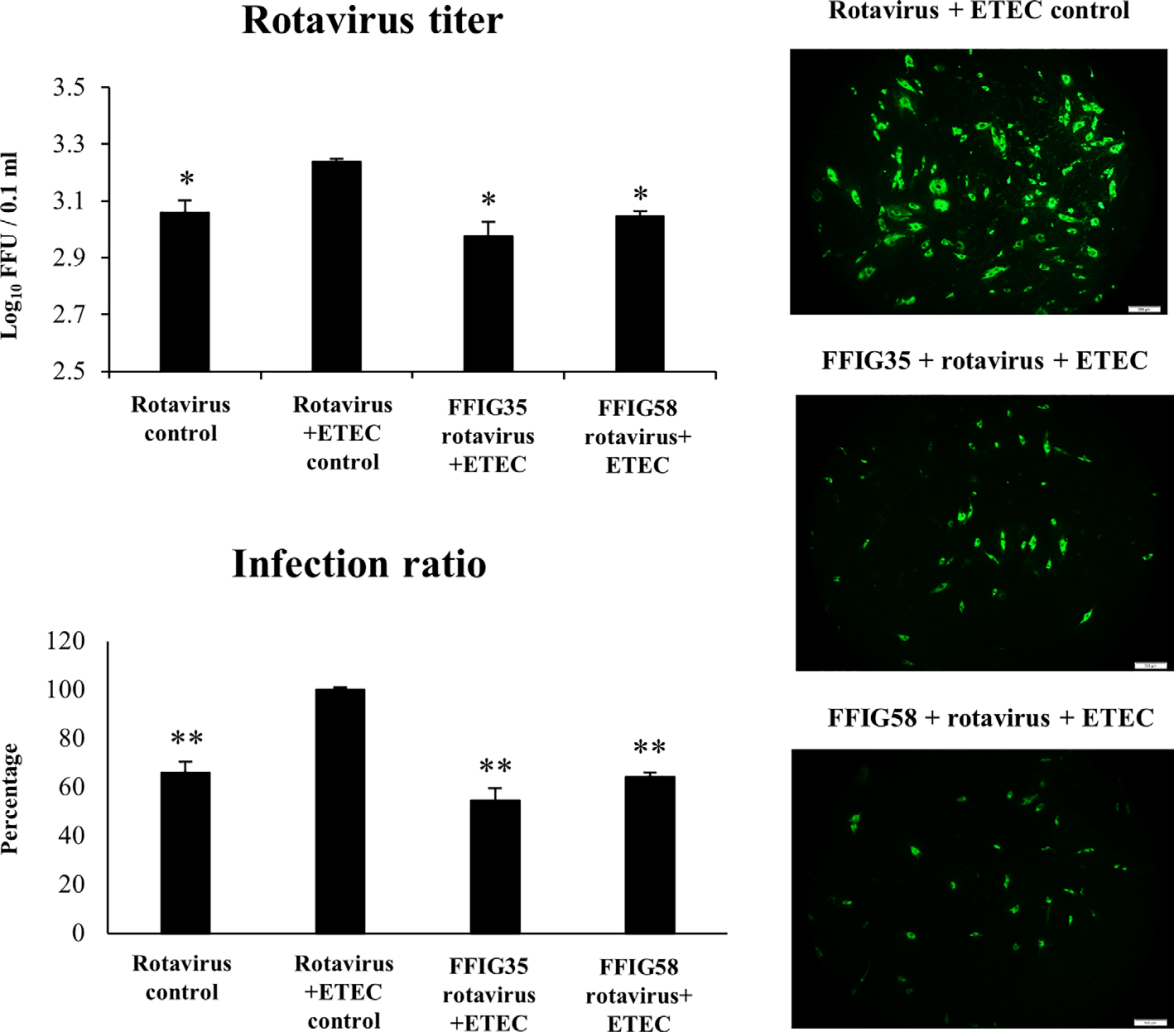
Effect of porcine *Ligilactobacillus salivarius* strains on the resistance of porcine intestinal epithelial (PIE) cells to enterotoxigenic *Escherichia coli* (ETEC) and rotavirus superinfection. PIE cells were stimulated with *L. salivarius* FFIG35 or FFIG58 isolated form the porcine gastrointestinal tract and then challenged with ETEC F6 and rotavirus. PIE cells with no lactobacilli treatment and challenged with ETEC and rotavirus or virus only were used for comparisons. Rotavirus infection was evaluated by immunofluorescence assay. The cells with specific green fluorescence in the cytoplasm were photographed by confocal laser microscopy after labeling with fluorescence anti-rotavirus antibody. The protective ability of lactobacilli was examined by calculating the virus titer and the infection ratio. The results represent data from three independent experiments. Values are means ± SD. Asterisks indicate significant differences when compared to the ETEC and rotavirus control group (*P < 0.05, **P < 0.01).

We next evaluated whether the co-administration of ETEC and rotavirus to PIE cells improved the viral replication and/or the severity of the inflammatory response. As shown in **Figure 7**, the co-administration of ETEC and rotavirus significantly enhanced the levels of virus titers as well as the infectious ratio when compared to PIE cells infected only with rotavirus. In addition, it was found that ETEC and rotavirus co-administration induced a higher expression of *IFN-λ3, PKR* (**Supplementary Figure 2**), *IL-6, IL-8*, and MCP-1 (**Supplementary Figure 3**) when compared to PIE cells infected only with the virus. ETEC and rotavirus co-administration also up-regulated *IFN-β* and *Mx1* in PIE cells, but the levels of expression were similar to those found in cells infected with rotavirus only (**Supplementary Figure 2**).

**Figure 8:**
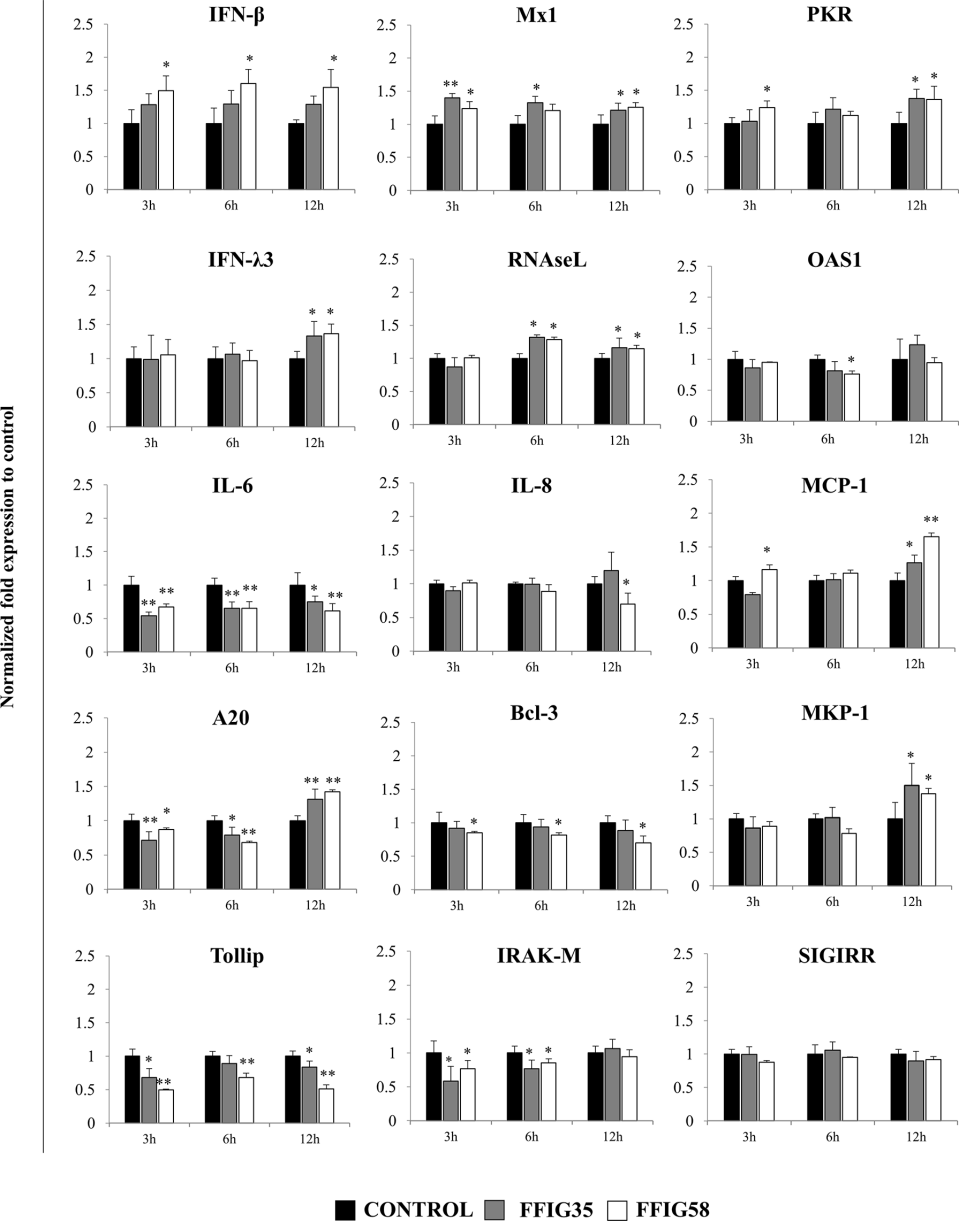
Effect of porcine *Ligilactobacillus salivarius* strains on the expression of immune factors in porcine intestinal epithelial (PIE) cells in response to enterotoxigenic *Escherichia coli* (ETEC) and rotavirus infection. PIE cells were stimulated with *L. salivarius* FFIG35 or FFIG58 isolated form the porcine gastrointestinal tract and then challenged with ETEC F6 and rotavirus. The expression of interferon (IFN)-β, IFN-λ3, protein kinase R (PKR), IFN-induced GTP-binding protein Mx1 (Mx1), ribonuclease L (RNAseL), 2’-5’-oligoadenylate synthetase 1 (OAS1), interleukin (IL)-6, IL-8 and monocyte chemoattractant protein 1 (MCP-1), zinc finger protein A20 (A20), B-cell lymphoma-3 (Bcl-3), Toll interacting protein (Tollip), interleukin-1 receptor-associated kinase M (IRAK-M), mitogen-activated protein kinase phosphatase-1 (MKP-1) and single immunoglobulin interleukin-1 related receptor (SIGIRR) were determined by RT-qPCR after 3, 6 or 12 hours of ETEC and rotavirus infection. PIE cells with no lactobacilli treatment and challenged with ETEC and rotavirus were used for comparisons. After normalization of genes with β-actin, the relative expression compared to the expression of each gene in the ETEC, and rotavirus control was calculated. The results represent data from three independent experiments at each time point. Values are means ± SD. Asterisks indicate significant differences when compared to the ETEC and rotavirus control group (*P < 0.05, **P < 0.01).

Of note, both FFIG35 and FFIG58 were capable of significantly reducing the rotavirus titers as well as infection ratio in PIE cells challenged with the virus and ETEC (**Figure 7**). Moreover, both strains were equally effective for improving the protection of cells against the viral infection. Interestingly, *L. salivarius* FFIG58-treated PIE cells had significantly higher levels of *IFN-β* after the ETEC/rotavirus challenge in all the time points evaluated when compared to FFIG35-treated and control PIE cells (**Figure 8**). Only the FFIG58 strain decreased the expression of *OAS1* in challenged PIE cells. On the other hand, both *L. salivarius* strains enhanced the expressions of *IFN-λ3, PKR, Mx1* and *RNAseL* at hour 12 post-ETEC/rotavirus challenge (**Figure 8**). In addition, both FFIG35 and FFIG58 decreased the expression of *IL-6* in all the time points evaluated while only *L. salivarius* FFIG58 down-regulated *IL-8* expression at hour 12. The expression levels of *MCP-1* were significantly increased by the FFIG58 strain at hours 3 and 12 while the FFIG35 strain increase this chemokine only at hour 12 (**Figure 8**).

When the negative regulators of the TLR signaling pathway were investigated after ETEC/rotavirus challenge, it was found that both FFIG35 and FFIG58 reduced the expressions of *Tollip* and *IRAK-M* (**Figure 8**). In addition, both *L. salivarius* strains reduced the expression of *A20* at hours 3 and 6 while they up-regulated this negative regulator at hour 12. No effect was observed when the expression of *SIGIRR* was analyzed while only the FFIG58 strain decreased *Bcl-3*. *MKP-1* was significantly increased at hour 12 post-ETEC/rotavirus challenge in both FFIG35- and FFIG58-treated PIE cells (**Figure 8**).

### Effect of Porcine *L. salivarius* FFIG58 on Poly(I:C) and Poly(I:C)/ETEC Challenges in Mice

**Figure 9:**
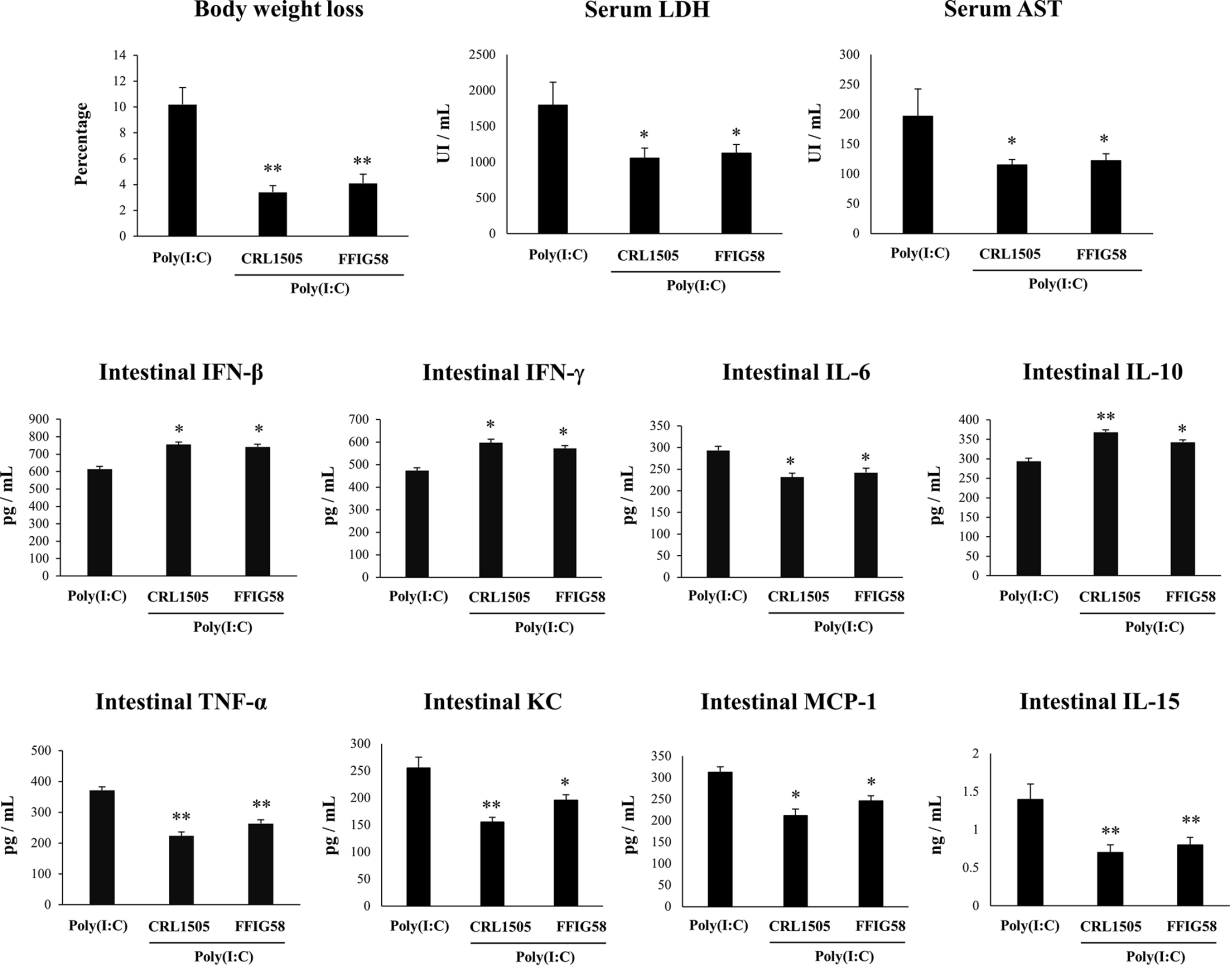
Immunomodulatory effect of porcine *Ligilactobacillus salivarius* FFIG58 in mice in response to poly(I:C) challenge. Mice were orally treated with *L. salivarius* FFIG58 or *Lacticaseibacillus rhamnosus* CRL1505 (10^8^ cells/mouse per day for 5 consecutive days) and then challenged by the intraperitoneal route with the viral molecular–associated pattern poly(I:C). Mice with no lactobacilli treatment and challenged with poly(I:C) were used as controls. Body weight loss, serum lactate dehydrogenase (LDH), serum aspartate aminotransferase (AST) and the intestinal levels of interferon (IFN)-β, IFN-γ, interleukin (IL)-6, IL-10, IL-15, tumor necrosis factor (TNF)-α, chemokine KC (or CXCL1), and monocyte chemoattractant protein 1 (MCP-1) were determined two days after the challenge with poly(I:C). The results represent data from three independent experiments (3 mice per group in each experiment). Values are means ± SD. Asterisks indicate significant differences when compared to the poly(I:C) control group (*P < 0.05, **P < 0.01).

In order to demonstrate the immunomodulatory abilities of *L. salivarius* FFIG58 *in vivo*, we used a mice model of poly(I:C)-triggered intestinal inflammation as we described previously (38, 39). In this experiments, the well characterized immunomodulatory strain *L. rhamnosus* CRL1505 (17, 30, 38, 41) was used for comparisons. Lactobacilli were administered to different groups of mice before poly(I:C) stimulation. We evaluated body weight loss to study the general health state of mice and the levels of serum LDH and AST to indirectly assess gastrointestinal damage after poly(I:C) challenge (**Figure 9**). The administration of poly(I:C) to mice significantly increased the body weight loss and the levels of serum LDH and AST as we described previously (38, 39). Interestingly, mice treated with *L. salivarius* FFIG58 had significantly lower percentages of body weight loss and levels of serum LDH and AST than controls. Moreover, the three parameters were not different from those observed in *L. rhamnosus* CRL1505-treated mice (**Figure 9**). We also evaluated the levels of inflammatory cytokines and chemokines as well as IL-10 in the intestinal tract of mice. Poly(I:C) challenge increased the levels of intestinal IFN-β, IFN-γ, TNF-α, IL-6, IL-15, KC, MCP-1, and IL-10 (**Figure 9**) when compared to basal levels (data not shown) in all the experimental groups. However, mice treated with the FFIG58 strain had concentrations of IFN-β, IFN-γ and IL-10 that were higher than controls. In addition, *L. salivarius* FFIG58 significantly reduced the levels of intestinal TNF-α, IL-6, IL-15, KC, and MCP-1 when compared to controls (**Figure 9**). The inflammatory cytokines and IL-10 concentrations in FFIG58-treated mice were not different from those observed in the *L. rhamnosus* CRL1505-treated group.

In order to evaluate the effect of *L. salivarius* FFIG58 on the resistance to ETEC inoculation after the intestinal viral inflammation, we developed a mice model of poly(I:C)/ETEC challenge. The comparison of ETEC and poly(I:C)/ETEC challenges in mice demonstrated a more severe disease caused by the bacterial pathogen after the damage of the intestinal mucosa by TLR3 activation (**Supplementary Figure 7**). In our hands, the percentages of body weight loss and the levels of serum LDH and AST were significantly higher in mice challenged with poly(I:C)/ETEC than in animals inoculated only with ETEC. In line with these findings, ETEC counts in jejunum, ileum, spleen, and liver were higher in the poly(I:C)/ETEC group than in ETEC-challenged mice. Moreover, the levels of intestinal IFN-β, IFN-γ, TNF-α, IL-6, IL-15, KC, and MCP-1 were higher in poly(I:C)/ETEC-treated mice than in the ETEC group (**Supplementary Figure 7**). Although the levels of intestinal IL-10 were higher in poly(I:C)/ETEC group than in ETEC-challenged mice, this difference was not statistically significant.

**Figure 10:**
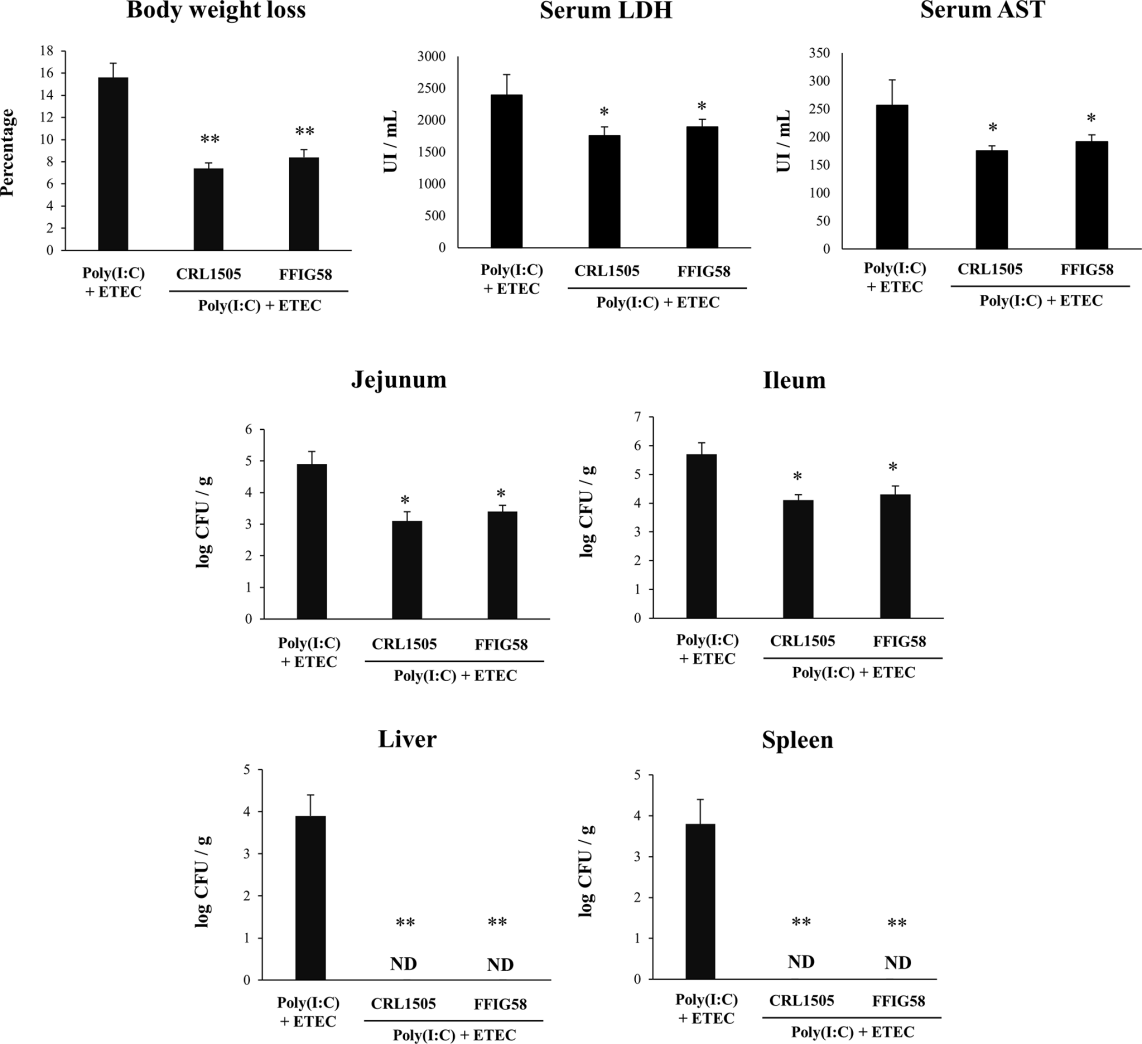
Immunomodulatory effect of porcine *Ligilactobacillus salivarius* FFIG58 in mice in response to poly(I:C) and enterotoxigenic *Escherichia coli* (ETEC) challenge. Mice were orally treated with *L. salivarius* FFIG58 or *Lacticaseibacillus rhamnosus* CRL1505 (10^8^ cells/mouse per day for 5 consecutive days) and then challenged by the intraperitoneal route with the viral molecular–associated pattern poly(I:C). Two days after poly(I:C) stimulation, mice were challenged orally with ETEC F4 strain (10^9^ cells). Mice with no lactobacilli treatment and challenged with poly(I:C) and ETEC were used as controls. Body weight loss, serum lactate dehydrogenase (LDH), serum aspartate aminotransferase (AST) and ETEC counts in jejunum, ileum, liver and spleen were determined two days after the challenge with ETEC. The results represent data from three independent experiments (3 mice per group in each experiment). Values are means ± SD. Asterisks indicate significant differences when compared to the poly(I:C)/ETEC control group (*P < 0.05, **P < 0.01).

**Figure 11:**
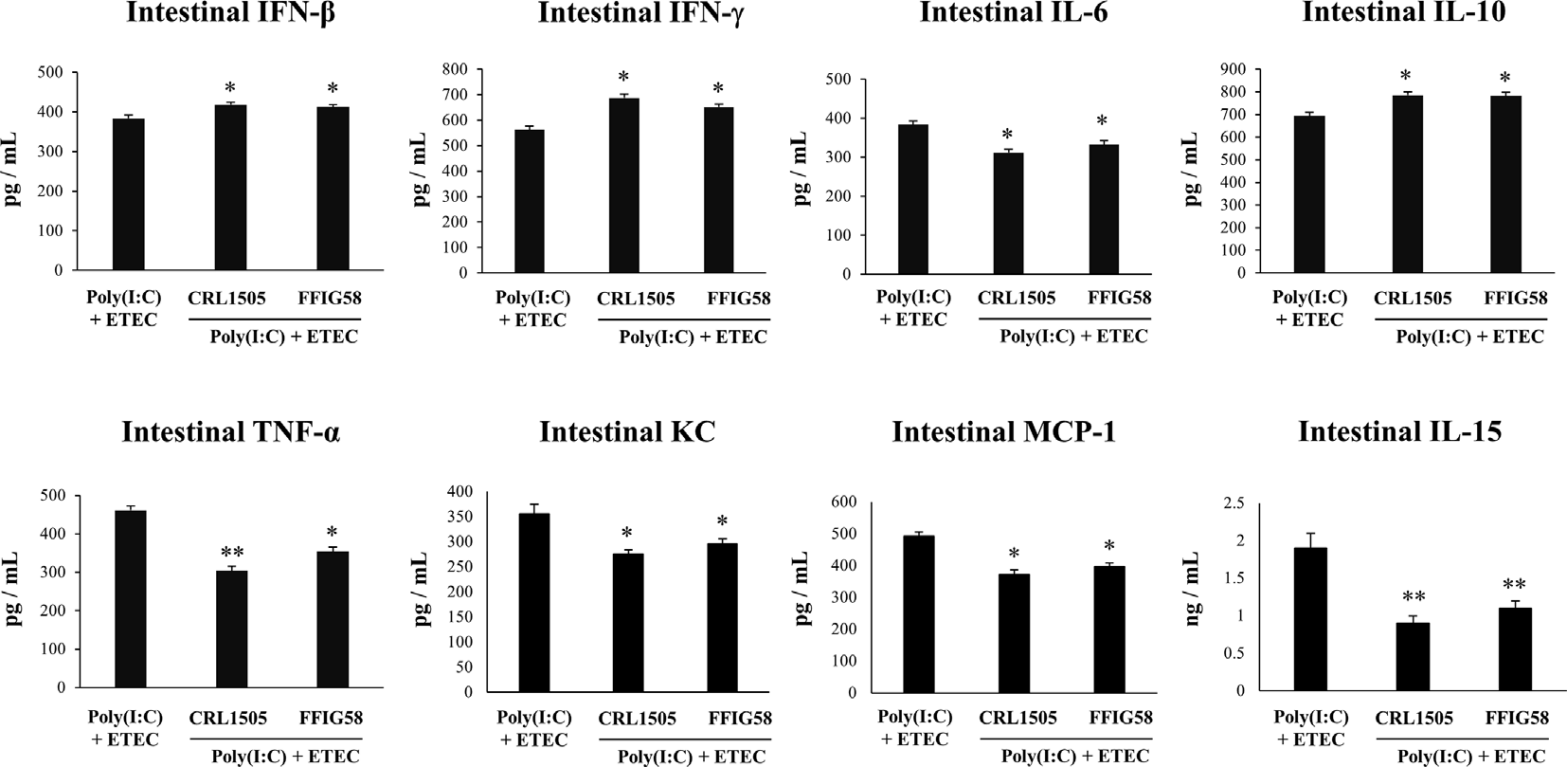
Immunomodulatory effect of porcine *Ligilactobacillus salivarius* FFIG58 in mice in response to poly(I:C) and enterotoxigenic *Escherichia coli* (ETEC) challenge. Mice were orally treated with *L. salivarius* FFIG58 or *Lacticaseibacillus rhamnosus* CRL1505 (10^8^ cells/mouse per day for 5 consecutive days) and then challenged by the intraperitoneal route with the viral molecular–associated pattern poly(I:C). Two days after poly(I:C) stimulation, mice were challenged orally with ETEC F4 strain (10^9^ cells). Mice with no lactobacilli treatment and challenged with poly(I:C) and ETEC were used as controls. The intestinal levels of interferon (IFN)-β, IFN-γ, interleukin (IL)-6, IL-10, IL-15, tumor necrosis factor (TNF)-α, chemokine KC (or CXCL1), and monocyte chemoattractant protein 1 (MCP-1) were determined two days after the challenge with ETEC. The results represent data from three independent experiments (3 mice per group in each experiment). Values are means ± SD. Asterisks indicate significant differences when compared to the poly(I:C)/ETEC control group (*P < 0.05, **P < 0.01).

Finally, we used the mice model of poly(I:C)/ETEC challenge to evaluate the effect of immunobiotics. Lactobacilli were administered to different groups of mice before poly(I:C) stimulation and ETEC challenge and the resistance to the infection was evaluated as shown in **Figure 10**. Both *L. salivarius* FFIG58 and *L. rhamnosus* CRL1505 were equally effective in reducing the percentages of body weight loss and the levels of serum LDH and AST in poly(I:C)/ETEC challenged mice. In addition, both FFIG58 and CRL1505 strains were able to significantly reduce the ETEC counts in jejunum and ileum when compared to controls. Furthermore, both lactobacilli treatments avoided the spread of the pathogen to spleen and liver (**Figure 10**). Mice treated with the FFIG58 or CRL1505 strains had concentrations of intestinal IFN-β, IFN-γ and IL-10 that were higher than controls (**Figure 11**). In addition, both *L. salivarius* FFIG58 and *L. rhamnosus* CRL1505 significantly reduced the levels of intestinal TNF-α, IL-6, IL-15, KC, and MCP-1 when compared to poly(I:C)/ETEC controls (**Figure 11**).

## Discussion

**Figure 12:**
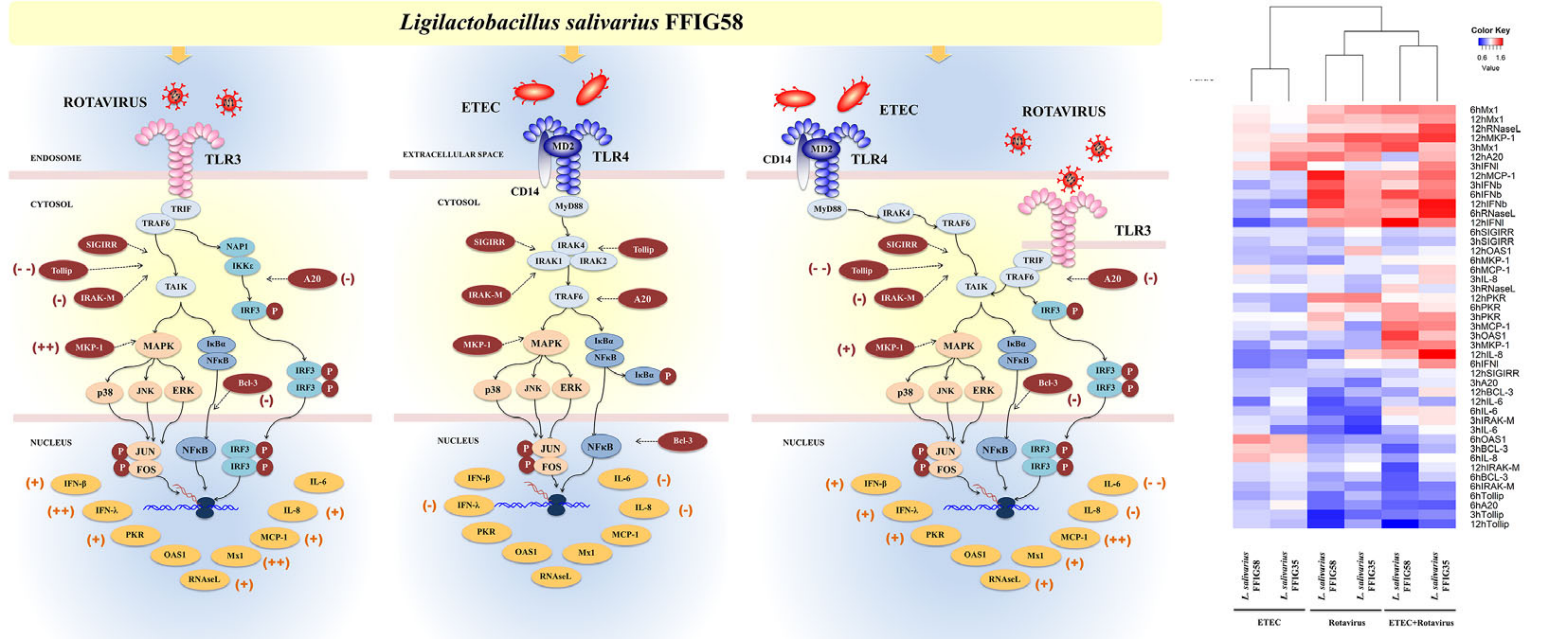
Immunomodulatory effect of porcine *Ligilactobacillus salivarius* FFIG58 on porcine intestinal epithelial (PIE) cells in response to rotavirus infection, enterotoxigenic *Escherichia coli* (ETEC) challenge and ETEC and rotavirus infection. The differential modulation of interferons (IFNs) and antiviral factors, inflammatory cytokines and chemokines and negative regulators of the Toll-like receptor (TLR) signaling pathway induced by *L. salivarius* FFIG58 are highlighted. The heat-map analysis compares the immunomodulatory effects of FFIG35 and FFIG58 strains. The heat-maps were constructed considering the fold expression changes in RT-qPCR data, related to non-lactobacilli treated controls. (+) moderate up-regulation, (++) heightened up-regulation, (-) moderate down-regulation, (–) heightened down-regulation. The changes indicated by (-) and (+) were calculated by considering the percentages.

It is well established that the interactions between microbes and intestinal epithelial cells (IECs) trigger cellular signaling pathways in the host cells that culminate in the generation of tolerance or effector immune responses, depending on the harmful potential of the microorganisms. Moreover, the interactions of luminal microbial products with IECs play a central role in determining the type of immune response triggered by intestinal microorganisms since IECs can influence the responses of mucosal immune cells. For these reasons, the study of the response of IECs to pathogenic, commensal or probiotic microorganisms has acquired great importance in recent years (17). In this regard, we demonstrated previously that the originally established porcine intestinal epithelial cell line (PIE cells) is a useful tool for studying innate immune responses triggered by PRRs and the influence of immunobiotic bacteria in those responses (30, 38). In addition, we showed that PIE cells are permissive to porcine rotavirus making them an excellent laboratory tool to study the epithelial antiviral immunity of the porcine host (32, 42). In this work, by using the *in vitro* PIE cell system we demonstrated that *L. salivarius* strains isolated from the gastrointestinal tract of wakame-fed pigs are able to differentially regulate the innate immune responses in porcine IECs. Two strains, *L. salivarius* FFIG35 and FFIG58 demonstrated remarkable immunomodulatory properties when their effect on the TLR3-triggered innate immune response in PIE cells was evaluated. The FFIG35 and FFIG58 strains were able to improve the resistance of PIE cells to rotavirus infection as well as to ETEC/rotavirus superinfection (**Figure 12**). Furthermore, studies in mice models of poly(I:C) and poly(I:C)/ETEC challenges demonstrated the capacity of *L. salivarius* FFIG58 to beneficially modulate intestinal immunity *in vivo*.

Rotavirus infects IECs located in the small intestinal proximal villi and its replication induce villous blunting leading to thinner and shorter villi. In addition, rotavirus promote villous atrophy since infected villi are covered by abnormal cuboidal epithelial cells (1, 2). Interestingly, differences in the innate immune responses triggered by rotavirus challenge in IECs have been associated to the age-dependent resistance. Studies in humans demonstrated that gastroenteritis is frequently found as a symptom in rotavirus-infected young infants while individuals older than 5 years are often asymptomatic (43). Moreover, the same study reported that the rotavirus susceptible group (children below 5 years) had significantly lower expression levels of TLR3 in the intestinal epithelium when compared to individuals between 5 to 20 years of age. Differences in the resistance against rotavirus infection were also found in adult versus young mice. While infants and neonatal mice are highly susceptible to rotavirus infection, adult individuals shed low numbers of viral particles and remain asymptomatic (43). Of note, TLR3 expression was low in the intestinal epithelium of suckling mice and was enhanced during the postnatal period. This TLR3 expression inversely correlated with the susceptibility of mice to rotavirus as shown by the differences in intestinal damage and viral shedding. Furthermore, it was reported that adult mice deficient in TLR3 or its adaptor molecule TRIF are highly susceptible to rotavirus infection than wild-type mice (43). Since rotavirus are ubiquitous, it is considered that every pig will experience the infection within its lifetime (1, 2). Similar to humans, it was shown that the prevalence of infection as well as the severity of disease depends on the age of the pig. Piglets that have not received proper passive immunity from the sow and young pigs without established immunity are more susceptible to rotavirus infections when compared to immunocompetent adults (1, 2). Those studies highlight the importance of developing strategies that help to modulate TLR3-mediated immunity in IECs of young individuals such as weaning pigs, in order to prevent severe cases of rotavirus infections.

In our hands, *L. salivarius* FFIG35 and FFIG58 were able to significantly reduce rotavirus replication in PIE cells when compared to control cells. This protective effect was associated to a differential modulation of the innate immune responses in PIE cells triggered by both the TLR3 agonist poly(I:C) and rotavirus. The FFIG35 and FFIG58 strains significantly up-regulated *IFN-β* and the antiviral factors *Mx1, RNAseL* and *PKR* in response to poly(I:C) stimulation or rotavirus challenge. It is known that rotavirus infection stimulates type I IFNs expression in IECs, particularly IFN-β, which elicit different types of responses on the same cell, neighbor IECs and surrounding immune cells (44–46). IFN-β induce an early antiviral gene expression in the gastrointestinal epithelium that is critical for rotavirus clearance. Among the hundreds of genes up-regulated by IFN-β, antiviral factors like Mx1, RNAseL and PKR have been shown to be indispensable for the elimination of rotavirus. Poly(I:C), similar to dsRNA from rotavirus can be recognized by IECs and activate PKR, which further improves the expression of IFN-responsive genes and pro-inflammatory cytokines and chemokines (47). Of note, the absence of the dsRNA recognition by PKR drives to a profound defect in the capacity of host cells to secrete IFN-β and to restrict the early replication of rotavirus (48). Furthermore, it was demonstrated that PKR induce the phosphorylation of the factor eIF2α that leads to the protein synthesis inhibition and the block in viral replication (49). On the other hand, OAS activates the latent RNAseL, which in turn degrade viral RNA molecules restricting their replication. In addition, the cleavage products produced by RNAseL activity in viral RNA molecules produce small RNA fractions that are recognized by RIG-I amplifying the production of IFN-β (50). It was reported that the OAS/RNaseL pathway is activated during rotavirus infection and is important for the defense of IECs (51). The myxovirus resistance proteins (Mx proteins) are GTPases that serve as intracellular restriction factors against virus replication. Among them, Mx1 was shown to inhibit viral replication by blocking the transcription of viral RNA (52), and this protein is considered an important antiviral factor for the protection of the IECs against rotavirus infection (53). The ability of *L. salivarius* FFIG58 to modulate the intestinal immune response triggered by TLR3 activation was further confirmed in a mice model. The oral administration of the FFIG58 strain to mice differentially regulated the intestinal cytokine profile in response to poly(I:C) stimulation as shown by the improved the production of IFN-β and IFN-γ, and the reduced levels of TNF-α, IL-6, IL-15, KC, and MCP-1. Furthermore, the immunomodulatory effect of the FFIG58 strain was comparable to the observed for the well characterized immunobiotic strain *L. rhamnosus* CRL1505 (38, 39), which was shown to improve the resistance of children to intestinal viral infections (54).

Interestingly, although *L. salivarius* FFIG35 and FFIG58 did not modify the expression of *IFN-λ3* in poly(I:C)-challenged PIE cells, both strains were able to significantly up-regulate this antiviral factor in PIE cells infected with rotavirus. Of note, *IFN-λ3* expression in poly(I:C)-challenged PIE cells was not different from the unchallenged PIE cells. In line with these findings, no variations in the intestinal levels of IFN-λ were observed in mice treated with poly(I:C) (data not shown). These results could be explained by the fact that the rotavirus is capable of activating not only TLR3 but in addition other PRRs expressed in the intestinal epithelium (1, 2). The multiple PRRs activation in IECs would culminate in the activation of several signaling pathways and in a cellular response somewhat different from that induced only by poly(I:C).

Type III IFNs (IFN-λ), similar to type I IFNs (IFN-α/β), are induced after the stimulation of PRRs, and the signal transduction events and the gene expression profiles are virtually indistinguishable one form the other (55, 56). However, IFN-α/β and IFN-λ differ strikingly regarding the spectrum of responsive cell types. IFN-λ is structurally different from IFN-α/β and signal thought a different heterodimeric receptor known as IL28R, which is constituted by the IL28Rα (or IFN-λR1) and the IL10Rβ chains (57). The receptors for IFN-α/β are expressed by all nucleated cells. In contrast, the functional receptors for IFN-λ are mainly expressed on epithelial cells (58). In addition, while IFN-α/β are secreted by a wide range of different immune and non-immune cell types upon stimulation, IFN-λ are primarily produced by epithelial cells and NK cells. Since rotavirus has developed multiple mechanisms to evade the antiviral actions of IFN-α/β (59), it is believed that the additional protection conferred by IFN-λ is of fundamental importance for a more efficient elimination of this viral pathogen from the intestinal mucosa. In support of this statement, it was reported that the infection of young mice with rotavirus increase the expression of both IFN-β and IFN-λ in IECs (60). Moreover, the treatment of mice with recombinant IFN-λ significantly reduced rotavirus replication in a more efficient way when compared to the type I IFN administration (60). In agreement with these findings, the elimination of IFN-λR1 induced higher levels of viral replication than wild-type mice while the knockout of the receptor for type I IFN induced the same affect but to a lesser degree (60). Of note, recent transcriptomic studies performed in the porcine intestinal epithelial cell line IPEC-J2 demonstrated that the pretreatment of these cells with IFN-λ3 or IFN-α resulted in a differential expression of antiviral genes. While IFN-α was capable of upregulating the expression of 134 genes, IFN-λ3 modified the expression of 983 genes (61). The results indicated that IFN-λ3 stimulated more robust antiviral signaling pathways, particularly the Jak-STAT pathway, when compared to IFN-α. These studies demonstrated the importance of the IFN-λ in the resistance against rotavirus infection and its role in complementing protection induced by IFN-α/β. These works also highlight the potential protective role that *L. salivarius* FFIG35 or FFIG58 could exert *in vivo* when administered to young pigs, through the modulation of the expression of both *IFN-λ3* and *IFN-β* and the upregulation of antiviral factors such as *OAS/RNAseL, PKR* and *Mx1* in IECs.

We have demonstrated previously that some immunobiotic bifidobacteria and lactobacilli are capable to modulate the innate immune response triggered by TLR4 activation in PIE cells (35–37). In this work, we also evaluated whether *L. salivarius* FFIG35 or FFIG58 were able to influence the TLR4-triggered response of PIE cells induced by ETEC challenge. Although the reduction of some inflammatory cytokines and chemokines were detected in lactobacilli-treated PIE cells, the effect of *L. salivarius* isolated from the gut of wakame-fed pigs was significantly lower than the observed for immunobiotic strains such as *Lactobacillus jensenii* TL2937 (35), *Bifidobacterium breve* M-16V, *Lactobacillus gasseri* MCC-1183 (36) or *Limosilactobacillus fermentum* UCO-979C (37). Interestingly, although a modest effect in the regulation of ETEC-induced innate immune response in PIE cells was observed for *L. salivarius* FFIG35 and FFIG58, both strains showed a remarkable effect in the modulation of ETEC/rotavirus superinfection (**Figure 12**). These findings may be related to the fact that the immune response of PIE cells to the ETEC/rotavirus superinfection appears to be dominated by the viral infection. In fact, the expression of *IFN-β, IFN-λ3*, antiviral factors, *IL-8* and *MCP-1* observed in PIE cells challenged with rotavirus showed a similar trend to the found in ETEC/rotavirus challenge, although it should be mentioned that the co-administration of ETEC significantly increased the values ​​of all these factors.

Studies in children demonstrated that there is a significant higher severity of diarrhea when the infection is produced by viral-bacterial mixed infections when compared to those induced by virus infections alone (62). These findings have been also reported in pigs. It was shown that rotavirus is the agent that more often is associated with the diarrhea in piglets. Of note, other pathogens could be detected in the outbreaks of rotavirus infections including pathogenic *E. coli* strains (63, 64). Studies in weaning of piglets demonstrated that the administration of pathogenic strains of *E. coli* significantly increased their susceptibility to a more severe rotavirus infection. While rotavirus was detected in control pigs, most of them did not experience severe diarrhea. In contrast, the groups of pigs exposed to pathogenic *E. coli* had a higher incidence of severe diarrhea (65). In fact, both the number of piglets developing diarrhea and the duration of the clinical signs were more prominent in the groups exposed to different serotypes of *E. coli.* In line with these findings, we demonstrated here that ETEC stimulation significantly increased the ability of rotavirus to replicate in PIE cells and a more potent inflammatory response was observed in ETEC/rotavirus challenge than in rotavirus alone. In addition, we observed that the challenge of mice with poly(I:C) and ETEC induced a significantly higher inflammatory response than the observed with poly(I:C) or ETEC alone. Moreover, the TLR3-mediated inflammation significantly increased the ability of ETEC to colonize the intestinal mucosa and to spread to liver and spleen. Then, the *in vitro* superinfection model in PIE cells and the *in vivo* mice model of poly(I:C)/ETEC challenge developed in this work could be useful to study therapeutic alternatives that help to reduce the most severe cases of diarrhea in young hosts.

Several studies have demonstrated the ability of immunobiotics to beneficially modulate the response to Gram-negative bacterial pathogens [reviewed (66)] or enteric virus [reviewed (17)] in pigs. However, few studies have demonstrated the ability of immunobiotic strains to protect against ETEC/rotavirus superinfection. In this regard, it was shown that the administration of *Bifidobacterium lactis* HN019 reduced the weanling diarrhea associated with rotavirus and pathogenic *E. coli* (67). The work demonstrated that HN019 administration reduced the severity of weanling diarrhea and allowed piglets to maintain a normal feed conversion efficiency. Although the beneficial effects were associated to an enhanced immune-mediated protection, detailed molecular immunological mechanisms were not investigated. Here, we demonstrated for the first time that immunobiotic lactobacilli differentially regulated the immune response of PIE cells to ETEC/rotavirus superinfection. Improved *IFN-λ*, *IFN-β* and antiviral factors as well as reduced expression of *IL-6* and *IL-8* were observed in PIE cells treated with FFIG58 or FFIG35 after the challenge with ETEC/rotavirus. In agreement, increased production of intestinal IFN-β and reduced TNF-α, IL-6, IL-15, and the mouse IL-8 homologue KC were detected in mice orally treated with *L. salivarius* FFIG58 and challenged with poly(I:C) and ETEC. This differential immune response induced by the FFIG58 strain correlated with an increased protection against ETEC inoculation. *L. salivarius* FFIG58 administration significantly reduced ETEC counts in jejunum and ileum and avoided the dissemination of the pathogen to liver and spleen, confirming *in vivo* the protective potential of porcine lactobacilli. Of note, the FFIG58 strain was able to increase the intestinal levels of IFN-γ and IL-10. The ability of probiotic lactobacilli to modulate the production of both IFN-γ and IL-10 have been attributed to their interactions with intestinal antigen presenting cells (37, 68, 69). Then, it could be speculated that *L. salivarius* FFIG58 would be also capable of modulating porcine intestinal antigen presenting cells. To evaluate the effect of the FFIG58 and FFIG35 strains on porcine intestinal antigen presenting cells could is an interesting topic for future research that could be of great value to understand the mechanisms of their immunological benefits.

We also demonstrated that immunobiotic lactobacilli improve the resistance to ETEC/rotavirus superinfection through the modulation of negative regulators of the TLR signaling pathway (**Figure 12**). TLR negative regulators play key roles in maintaining intestinal hemostasis and regulating the immune responses against pathogens. Among them, the protein A20 is capable to terminate TLR signaling that result in the inhibition of NF-κB activation and the expression of inflammatory factors (70). In addition, A20 is able to interact with the complex IKKi/IKKϵ and suppress the dimerization of IRF3 after the engagement of TLR3 by viral dsRNA (71). In this way, the A20 protein induce the suppression of the IFN-mediated immune responses. For this reason, it is considered that treatments capable of avoiding the increase of A20 during viral infections could help to enhance the innate immune response mediated by IFNs. In this regard, studies evaluating the effect of probiotic microorganisms in the modulation of poly(I:C)-induced immune response in HT-29 cells demonstrated that probiotics significantly reduced A20 expression levels potentiating the IFN response (72). Moreover, we have previously demonstrated that *B. infantis* MCC12, *B. breve* MCC1274 (32), *L. rhamnosus* CRL1505, *Lactiplantibacillus plantarum* CRL1506 (30), and *L. plantarum* MPL16 (38) significantly diminished A20 expression in PIE cells in the context of poly(I:C) stimulation or rotavirus infection, which was in line with the capacity of these strains to improve IRF3 activation and IFN-β production. Here, we observed a similar effect for *L. salivarius* FFIG35 and FFIG58 in both poly(I:C)- and rotavirus-challenged PIE cells. Furthermore, the FFIG35 and FFIG58 strains were also capable of reducing *A20* expression in ETEC/rotavirus-challenged PIE cells.

*L. salivarius* FFIG35 and FFIG58 were also capable of reducing the expression levels of *Tollip, IRAK-M* and *Bcl-3* in PIE cells challenged with both rotavirus and ETEC/rotavirus. The expression of these three negative regulators of the TLR signaling in the intestinal epithelium has been associated to the tolerance in steady conditions. It was demonstrated that knockdown of Tollip in Caco-2 epithelial cells led to exaggerated NF-κB activity and pro-inflammatory cytokine secretion (73). Moreover, the same work reported that Tollip-deficient mice had increased intestinal permeability and augmented epithelial apoptosis when compared with wild-type controls. The Bcl-3 protein is an inhibitor of NF-κB, which was proposed to be a key player in the process of LPS tolerance (74). On the other hand, it was reported that IRAK-M expression is induced upon LPS stimulation, and endotoxin tolerance is diminished in IRAK-M-deficient cells (75). IRAK-M prevents the formation of the TRAF6/IRAK-1 complex, which initiate NF-κB and MAPK signaling pathways (76). Overexpression of these TLR negative regulators impairs TLR-triggered NF-κB and MAPK signaling pathways (77). In fact, it was shown that the exposure of IECs to TLR ligands creates a hyporesponsive state to a second challenge with the same or another TLR ligand, impairing the expression of pro-inflammatory factors. Then, during the earlier steps of the innate immune response against pathogens, these TLR negative regulators should be down-regulated in order to allow the efficient generation of inflammatory responses to eliminate the pathogenic microbes from mucosal surfaces. Of note, *L. salivarius* FFIG35 and FFIG58 increased the expression of *MKP-1*, which is a TLR negative regulator that plays an important role in the inhibition of pro-inflammatory responses by the inactivation of the MAPK pathway (78, 79). Thus, the differential regulation in the TLR negative regulators expression by *L. salivarius* FFIG35 or FFIG58 in PIE cells may be important for establishing IRF3-, NF-κB- and MAPK-mediated innate immune responses against rotavirus and ETEC/rotavirus, allowing an efficient induction of an antiviral state in the intestinal epithelium, the generation of signals that improve the recruitment and activation of immune cells and the protection against the inflammatory-mediated damage. Our *in vivo* studies in mice indicated that *L. salivarius* FFIG58 was indeed capable of stimulating the intestinal antiviral immune response and reducing inflammatory cytokines, increasing the protection against the poly(I:C)/ETEC challenge. To find out whether these effects can be achieved *in vivo* by the oral administration of the FFIG35 or FFIG58 strains to pigs is an important topic for future near research.

## Conclusions

We demonstrated here that *L. salivarius* strains isolated from the gastrointestinal tract of wakame-fed pigs have immunomodulatory properties in PIE cells and are capable of modulating TLR3-mediated immune responses. Moreover, we showed that *L. salivarius* FFIG35 and FFIG58 were able to improve the resistance of PIE cells to rotavirus infection as well as to ETEC/rotavirus superinfection. In addition, experiments in mice models of poly(I:C) and poly(I:C)/ETEC challenges confirmed *in vivo* the immunomodulatory abilities of the FFIG58 strain. The results of this work allow us to hypothesize that the FFIG35 or FFIG58 strains could be used for the development of highly efficient functional feed to improve immune health status and reduce the severity of intestinal infections and superinfections in weaned piglets. Interestingly, some studies have demonstrated that the individual administration of probiotic strains such as *L. rhamnosus* GG exert protective activity against rotavirus infection in pigs (19, 20). Of note, when a mixture of probiotic strains such as *L. rhamnosus* GG plus *B. lactis* Bb12 or *L. rhamnosus* GG plus *E. coli* Nissle 1917 were administered to pigs, a synergistic effect in the reduction of the duration and severity of rotavirus diarrhea was observed (21–23). Then, in addition to the *in vivo* evaluation of the individual strains, a combination of *L. salivarius* FFIG35 and FFIG58 should be also performed in *in vivo* porcine experiments to find the most efficient way to use these immunomodulatory strains in the generation of a new immunobiotic feed to help in the prevention of infections in the porcine host.

## Data Availability Statement

The raw data supporting the conclusions of this article will be made available by the authors, without undue reservation.

## Ethics Statement

The animal study was reviewed and approved by Ethical Committee of Animal Care at CERELA, Argentina.

## Author Contributions

JV and HK designed the study and manuscript writing. YI, SKi, TM, SA, BZ, AM, and WI-O did the laboratory work. LA and MI did the statistical analysis. TN, TT, HU, HA, HT, SKu, JV, and HK contributed to data analysis and interpretation. All authors contributed to the article and approved the submitted version.

## Funding

This study was supported by grants from the project of NARO Bio-oriented Technology Research Advancement Institution (Research Program on the Development of Innovative Technology, No. 01002A), by a Grant-in-Aid for Scientific Research (A) (19H00965) from the Japan Society for the Promotion of Science (JSPS), by Japan Racing Association (JRA) Livestock Industry Promotion Project, and by the Association for Research on Lactic Acid Bacteria to HK. This study was also supported by ANPCyT–FONCyT Grant PICT-2016-0410 to JV, and by Tohoku University Research Program “Frontier Research in Duo” (FRiD) to SK. This work was also supported by grants for Scientific Research on Innovative Areas from the Ministry of Education, Culture, Science, Sports, and Technology (MEXT) of Japan (16H06429, 16K21723, and 16H06435) and Grant-in-Aid for Challenging Research (Exploratory, 19K22300) to HT, and by JSPS Core-to-Core Program, A. Advanced Research Networks entitled Establishment of international agricultural immunology research-core for a quantum improvement in food safety, and by AMED Grant Number JP21zf0127001. MAI was supported by JSPS (Postdoctoral Fellowship for Foreign Researchers, Program No. 18F18081). M.T. was supported by Tohoku University Global Hagi Scholarship.

## Conflict of Interest

The authors declare that the research was conducted in the absence of any commercial or financial relationships that could be construed as a potential conflict of interest.

## Supplementary Material

Supplementary Figure 1 Effect of porcine *Ligilactobacillus salivarius* strains on the expression of negative regulators of the Toll-like receptor (TLR) signaling pathway in porcine intestinal epithelial (PIE) cells in response to TLR3 activation. PIE cells were stimulated with *L. salivarius* FFIG35, FFIG56 or FFIG58 isolated form the porcine gastrointestinal tract and then challenged with poly(I:C) to activate TLR3. The expression of mitogen-activated protein kinase phosphatase-1 (MKP-1) and single immunoglobulin interleukin-1 related receptor (SIGIRR) were determined by RT-qPCR after 3, 6 or 12 hours of TLR3 activation. PIE cells with no challenge (basal control) or stimulated only with poly(I:C) (poly(I:C) control) were used for comparisons. After normalization of genes with β-actin, the relative expression compared to the expression of each gene in the poly(I:C) control was calculated. The results represent data from three independent experiments. Values are means ± SD. Asterisks indicate significant differences when compared to the poly(I:C) control group (*P < 0.05, **P < 0.01).

Supplementary Figure 2 Effect of rotavirus infection, enterotoxigenic *Escherichia coli* (ETEC) challenge and ETEC and rotavirus infection on the expression of interferons (IFNs) and antiviral factors in porcine intestinal epithelial (PIE) cells. PIE cells were challenged with rotavirus, ETEC or ETEC F6 and rotavirus. The expression of IFN-β, IFN-λ3, protein kinase R (PKR), IFN-induced GTP-binding protein Mx1 (Mx1), ribonuclease L (RNAseL) and 2’-5’-oligoadenylate synthetase 1 (OAS1) were determined by RT-qPCR before challenges (hour 0) and after 3, 6 or 12 hours of rotavirus, ETEC or ETEC and rotavirus infection. After normalization of genes with β-actin, the relative expression compared to the expression of each gene in the controls at time 0 was calculated. The results represent data from three independent experiments at each time point. Values are means ± SD. Asterisks indicate significant differences when compared to the time 0 within the same group (*P < 0.05, **P < 0.01).

Supplementary Figure 3 Effect of rotavirus infection, enterotoxigenic *Escherichia coli* (ETEC) challenge and ETEC and rotavirus infection on the expression of inflammatory cytokines and chemokines in porcine intestinal epithelial (PIE) cells. PIE cells were challenged with rotavirus, ETEC F6 or ETEC and rotavirus. The expression of interleukin (IL)-6, IL-8 and monocyte chemoattractant protein 1 (MCP-1) were determined by RT-qPCR before challenges (hour 0) and after 3, 6 or 12 hours of rotavirus, ETEC or ETEC and rotavirus infection. After normalization of genes with β-actin, the relative expression compared to the expression of each gene in the controls at time 0 was calculated. The results represent data from three independent experiments at each time point. Values are means ± SD. Asterisks indicate significant differences when compared to the time 0 within the same group (*P < 0.05, **;P < 0.01).

Supplementary Figure 4 Effect of porcine *Ligilactobacillus salivarius* strains on the expression of interferons (IFNs) and antiviral factors in porcine intestinal epithelial (PIE) cells in response to enterotoxigenic *Escherichia coli* (ETEC) challenge. PIE cells were stimulated with *L. salivarius* FFIG35 or FFIG58 isolated form the porcine gastrointestinal tract and then challenged with ETEC F6. The expression of IFN-β, IFN-λ3, protein kinase R (PKR), IFN-induced GTP-binding protein Mx1 (Mx1), ribonuclease L (RNAseL) and 2’-5’-oligoadenylate synthetase 1 (OAS1) were determined by qPCR after 3, 6 or 12 hours of ETEC challenge. PIE cells with no lactobacilli treatment and challenged with ETEC were used for comparisons. After normalization of genes with β-actin, the relative expression compared to the expression of each gene in the ETEC control was calculated. The results represent data from three independent experiments at each time point. Values are means ± SD. Asterisks indicate significant differences when compared to the ETEC control group (*P < 0.05).

Supplementary Figure 5 Effect of porcine *Ligilactobacillus salivarius* strains on the expression of inflammatory cytokines and chemokines in porcine intestinal epithelial (PIE) cells in response to enterotoxigenic *Escherichia coli* (ETEC) challenge. PIE cells were stimulated with *L. salivarius* FFIG35 or FFIG58 isolated form the porcine gastrointestinal tract and then challenged with ETEC F6. The expression of interleukin (IL)-6, IL-8 and monocyte chemoattractant protein 1 (MCP-1) were determined by RT-qPCR after 3, 6 or 12 hours of ETEC challenge. PIE cells with no lactobacilli treatment and challenged with ETEC were used for comparisons. After normalization of genes with β-actin, the relative expression compared to the expression of each gene in the ETEC control was calculated. The results represent data from three independent experiments at each time point. Values are means ± SD. Asterisks indicate significant differences when compared to the ETEC control group (*P < 0.05).

Supplementary Figure 6 Effect of porcine *Ligilactobacillus salivarius* strains on the expression of negative regulators of the Toll-like receptor (TLR) signaling pathway in porcine intestinal epithelial (PIE) cells in response to enterotoxigenic *Escherichia coli* (ETEC) challenge. PIE cells were stimulated with *L. salivarius* FFIG35 or FFIG58 isolated form the porcine gastrointestinal tract and then challenged with ETEC F6. The expression of zinc finger protein A20 (A20), B-cell lymphoma-3 (Bcl-3), Toll interacting protein (Tollip), interleukin-1 receptor-associated kinase M (IRAK-M), mitogen-activated protein kinase phosphatase-1 (MKP-1) and single immunoglobulin interleukin-1 related receptor (SIGIRR) were determined by RT-qPCR after 3, 6 or 12 hours of ETEC challenge. PIE cells with no lactobacilli treatment and challenged with ETEC were used for comparisons. After normalization of genes with β-actin, the relative expression compared to the expression of each gene in the ETEC control was calculated. The results represent data from three independent experiments at each time point. Values are means ± SD. No significant differences were found when lactobacilli-treated cells were compared to the ETEC control group.

Supplementary Figure 7 Response of mice to poly(I:C) and enterotoxigenic *Escherichia coli* (ETEC) challenges. Mice were challenged by the intraperitoneal route with the viral molecular–associated pattern poly(I:C). Two days after poly(I:C) stimulation, mice were challenged orally with ETEC F4 strain (10^9^ cells). Mice infected with ETEC only were used for comparisons. Body weight loss, serum lactate dehydrogenase (LDH), serum aspartate aminotransferase (AST), ETEC counts in jejunum, ileum, liver and spleen and the intestinal levels of interferon (IFN)-β, IFN-γ, interleukin (IL)-6, IL-10, IL-15, tumor necrosis factor (TNF)-α, chemokine KC (or CXCL1), and monocyte chemoattractant protein 1 (MCP-1) were determined two days after the challenge with ETEC. The results represent data from three independent experiments (3 mice per group in each experiment). Values are means ± SD. Asterisks indicate significant differences when compared to the ETEC control group (*P < 0.05, **P < 0.01).
