# Supplementary figures and images for: Ligilactobacillus salivarius Strains Isolated From the Porcine Gut Modulate Innate Immune Responses in Epithelial Cells and Improve Protection Against Intestinal Viral-Bacterial Superinfection
Source: Front Immunol. 2021 Jun 7;12:652923. doi: 10.3389/fimmu.2021.652923 (PMC8215365; doi:10.3389/fimmu.2021.652923)

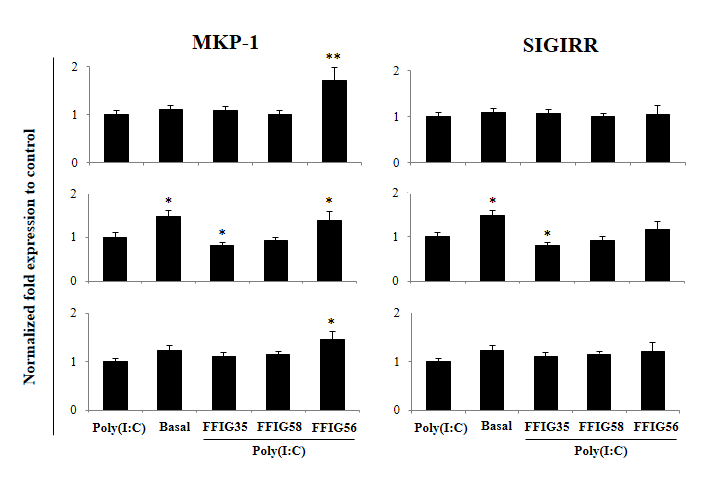

Supplement: Supplementary Figure 1 — Effect of porcine Ligilactobacillus salivarius strains on the expression of negative regulators of the Toll-like receptor (TLR) signaling pathway in porcine intestinal epithelial (PIE) cells in response to TLR3 activation. PIE cells were stimulated with L. salivarius FFIG35, FFIG56 or FFIG58 isolated form the porcine gastrointestinal tract and then challenged with poly(I:C) to activate TLR3. The expression of mitogen-activated protein kinase phosphatase-1 (MKP-1) and single immunoglobulin interleukin-1 related receptor (SIGIRR) were determined by RT-qPCR after 3, 6 or 12 hours of TLR3 activation. PIE cells with no challenge (basal control) or stimulated only with poly(I:C) (poly(I:C) control) were used for comparisons. After normalization of genes with β-actin, the relative expression compared to the expression of each gene in the poly(I:C) control was calculated. The results represent data from three independent experiments. Values are means ± SD. Asterisks indicate significant differences when compared to the poly(I:C) control group (*P < 0.05, **P < 0.01). [file Image_1.tif]

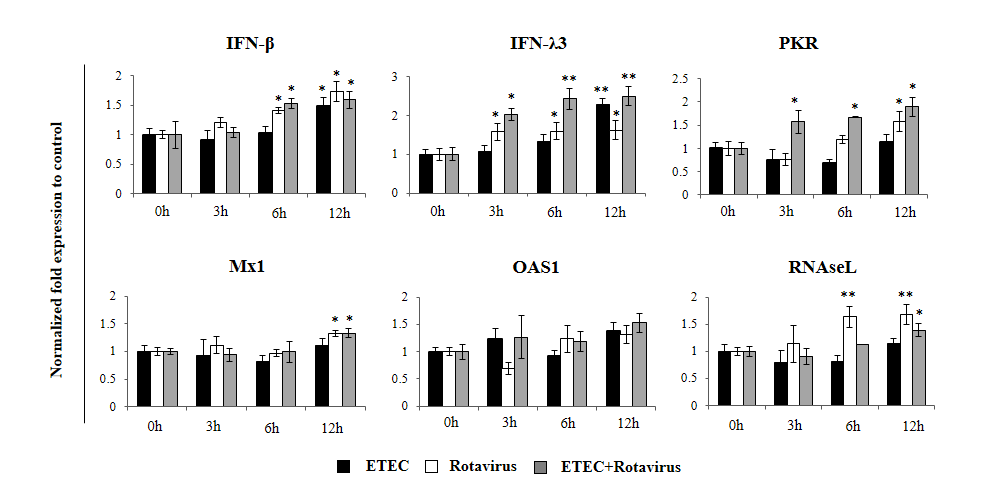

Supplement: Supplementary Figure 2 — Effect of rotavirus infection, enterotoxigenic Escherichia coli (ETEC) challenge and ETEC and rotavirus infection on the expression of interferons (IFNs) and antiviral factors in porcine intestinal epithelial (PIE) cells. PIE cells were challenged with rotavirus, ETEC or ETEC F6 and rotavirus. The expression of IFN-β, IFN-λ3, protein kinase R (PKR), IFN-induced GTP-binding protein Mx1 (Mx1), ribonuclease L (RNAseL) and 2’-5’-oligoadenylate synthetase 1 (OAS1) were determined by RT-qPCR before challenges (hour 0) and after 3, 6 or 12 hours of rotavirus, ETEC or ETEC and rotavirus infection. After normalization of genes with β-actin, the relative expression compared to the expression of each gene in the controls at time 0 was calculated. The results represent data from three independent experiments at each time point. Values are means ± SD. Asterisks indicate significant differences when compared to the time 0 within the same group (*P < 0.05, **P < 0.01). [file Image_2.tif]

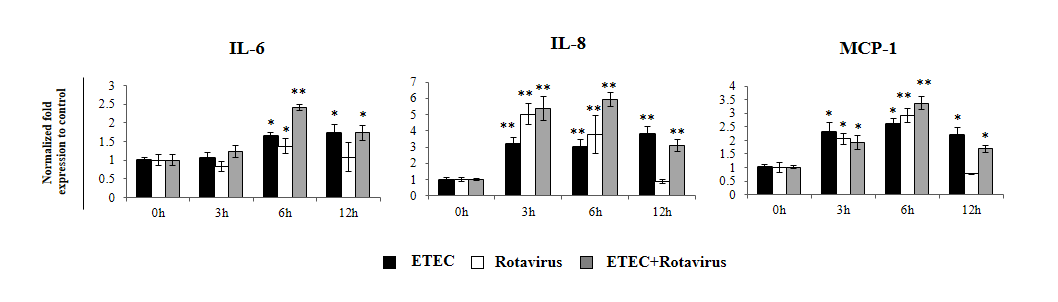

Supplement: Supplementary Figure 3 — Effect of rotavirus infection, enterotoxigenic Escherichia coli (ETEC) challenge and ETEC and rotavirus infection on the expression of inflammatory cytokines and chemokines in porcine intestinal epithelial (PIE) cells. PIE cells were challenged with rotavirus, ETEC F6 or ETEC and rotavirus. The expression of interleukin (IL)-6, IL-8 and monocyte chemoattractant protein 1 (MCP-1) were determined by RT-qPCR before challenges (hour 0) and after 3, 6 or 12 hours of rotavirus, ETEC or ETEC and rotavirus infection. After normalization of genes with β-actin, the relative expression compared to the expression of each gene in the controls at time 0 was calculated. The results represent data from three independent experiments at each time point. Values are means ± SD. Asterisks indicate significant differences when compared to the time 0 within the same group (*P < 0.05, **;P < 0.01). [file Image_3.tif]

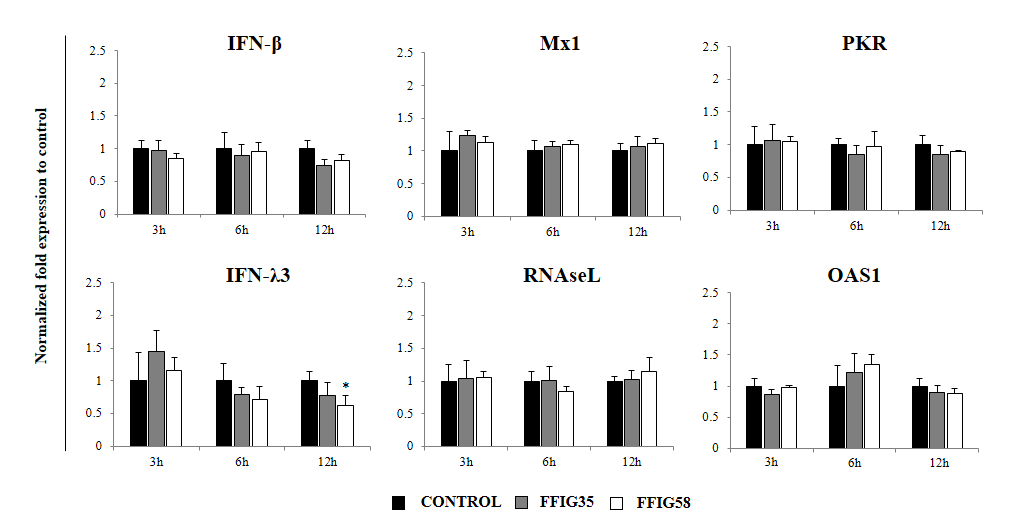

Supplement: Supplementary Figure 4 — Effect of porcine Ligilactobacillus salivarius strains on the expression of interferons (IFNs) and antiviral factors in porcine intestinal epithelial (PIE) cells in response to enterotoxigenic Escherichia coli (ETEC) challenge. PIE cells were stimulated with L. salivarius FFIG35 or FFIG58 isolated form the porcine gastrointestinal tract and then challenged with ETEC F6. The expression of IFN-β, IFN-λ3, protein kinase R (PKR), IFN-induced GTP-binding protein Mx1 (Mx1), ribonuclease L (RNAseL) and 2’-5’-oligoadenylate synthetase 1 (OAS1) were determined by qPCR after 3, 6 or 12 hours of ETEC challenge. PIE cells with no lactobacilli treatment and challenged with ETEC were used for comparisons. After normalization of genes with β-actin, the relative expression compared to the expression of each gene in the ETEC control was calculated. The results represent data from three independent experiments at each time point. Values are means ± SD. Asterisks indicate significant differences when compared to the ETEC control group (*P < 0.05). [file Image_4.tif]

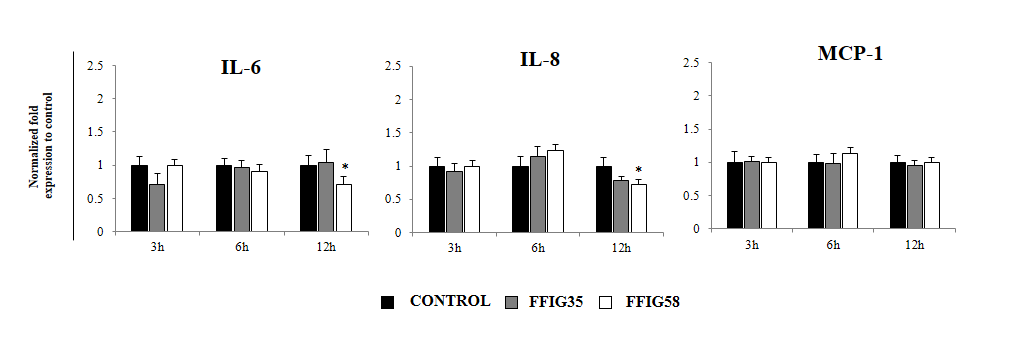

Supplement: Supplementary Figure 5 — Effect of porcine Ligilactobacillus salivarius strains on the expression of inflammatory cytokines and chemokines in porcine intestinal epithelial (PIE) cells in response to enterotoxigenic Escherichia coli (ETEC) challenge. PIE cells were stimulated with L. salivarius FFIG35 or FFIG58 isolated form the porcine gastrointestinal tract and then challenged with ETEC F6. The expression of interleukin (IL)-6, IL-8 and monocyte chemoattractant protein 1 (MCP-1) were determined by RT-qPCR after 3, 6 or 12 hours of ETEC challenge. PIE cells with no lactobacilli treatment and challenged with ETEC were used for comparisons. After normalization of genes with β-actin, the relative expression compared to the expression of each gene in the ETEC control was calculated. The results represent data from three independent experiments at each time point. Values are means ± SD. Asterisks indicate significant differences when compared to the ETEC control group (*P < 0.05). [file Image_5.tif]

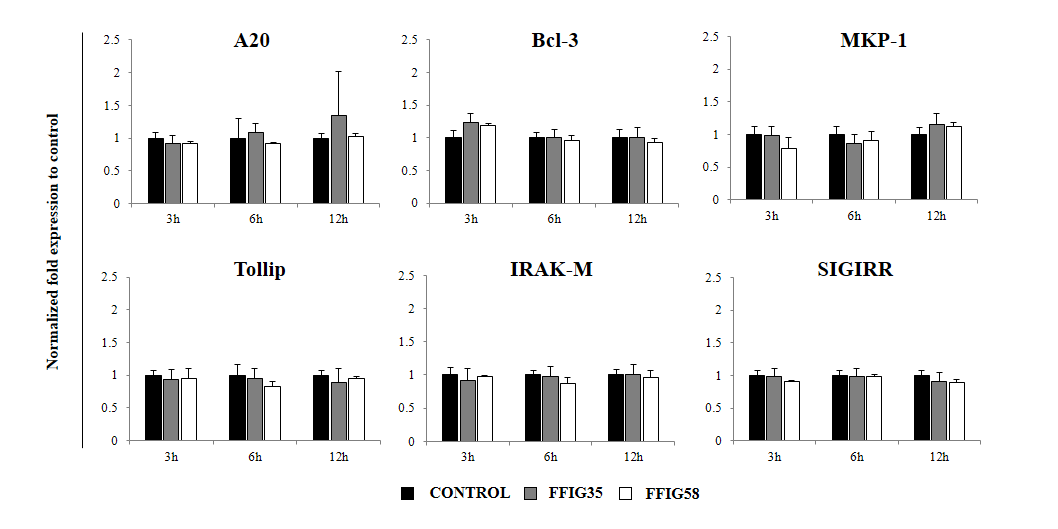

Supplement: Supplementary Figure 6 — Effect of porcine Ligilactobacillus salivarius strains on the expression of negative regulators of the Toll-like receptor (TLR) signaling pathway in porcine intestinal epithelial (PIE) cells in response to enterotoxigenic Escherichia coli (ETEC) challenge. PIE cells were stimulated with L. salivarius FFIG35 or FFIG58 isolated form the porcine gastrointestinal tract and then challenged with ETEC F6. The expression of zinc finger protein A20 (A20), B-cell lymphoma-3 (Bcl-3), Toll interacting protein (Tollip), interleukin-1 receptor-associated kinase M (IRAK-M), mitogen-activated protein kinase phosphatase-1 (MKP-1) and single immunoglobulin interleukin-1 related receptor (SIGIRR) were determined by RT-qPCR after 3, 6 or 12 hours of ETEC challenge. PIE cells with no lactobacilli treatment and challenged with ETEC were used for comparisons. After normalization of genes with β-actin, the relative expression compared to the expression of each gene in the ETEC control was calculated. The results represent data from three independent experiments at each time point. Values are means ± SD. No significant differences were found when lactobacilli-treated cells were compared to the ETEC control group. [file Image_6.tif]

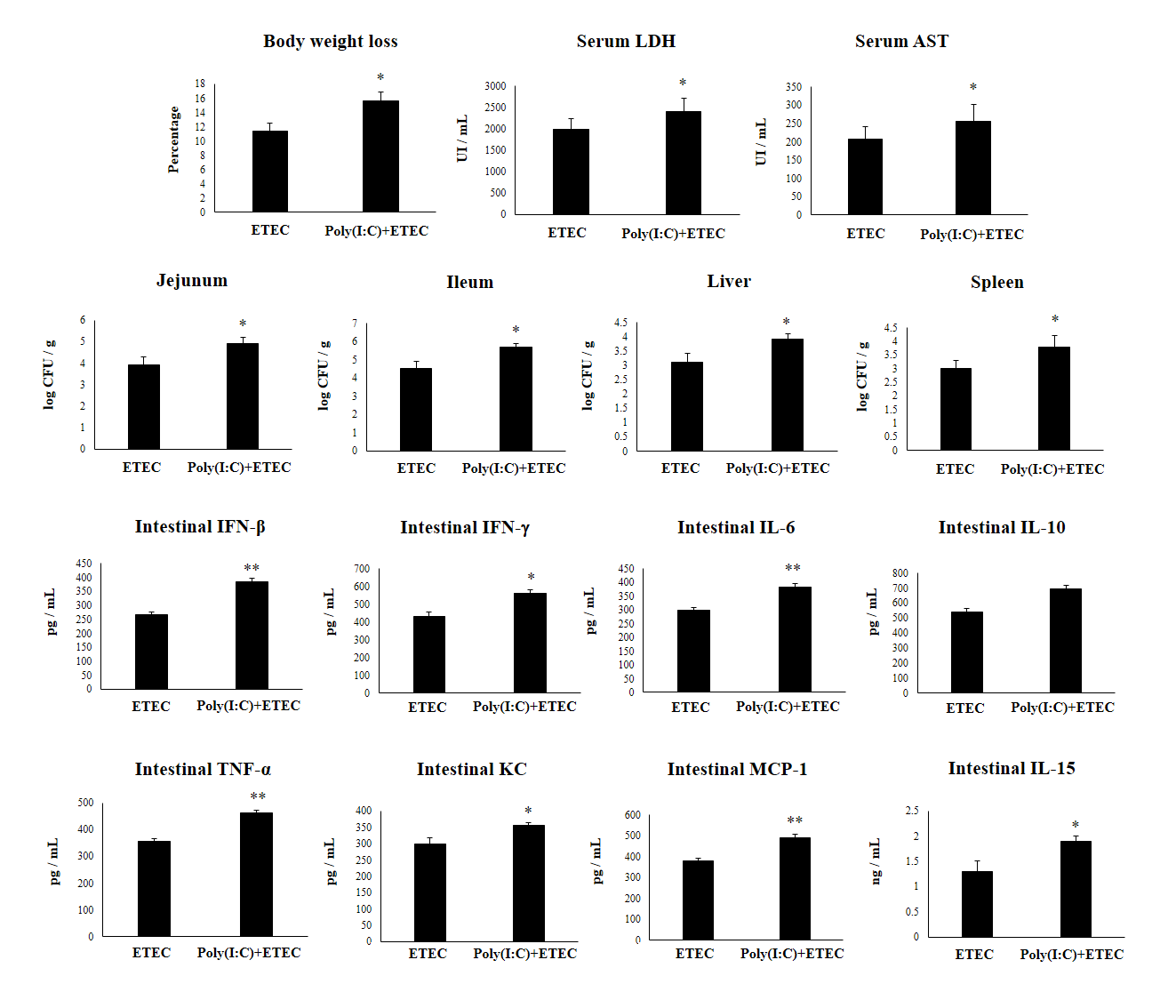

Supplement: Supplementary Figure 7 — Response of mice to poly(I:C) and enterotoxigenic Escherichia coli (ETEC) challenges. Mice were challenged by the intraperitoneal route with the viral molecular–associated pattern poly(I:C). Two days after poly(I:C) stimulation, mice were challenged orally with ETEC F4 strain (109 cells). Mice infected with ETEC only were used for comparisons. Body weight loss, serum lactate dehydrogenase (LDH), serum aspartate aminotransferase (AST), ETEC counts in jejunum, ileum, liver and spleen and the intestinal levels of interferon (IFN)-β, IFN-γ, interleukin (IL)-6, IL-10, IL-15, tumor necrosis factor (TNF)-α, chemokine KC (or CXCL1), and monocyte chemoattractant protein 1 (MCP-1) were determined two days after the challenge with ETEC. The results represent data from three independent experiments (3 mice per group in each experiment). Values are means ± SD. Asterisks indicate significant differences when compared to the ETEC control group (*P < 0.05, **P < 0.01). [file Image_7.tif]
