# Supplementary material for: Ligilactobacillus salivarius Strains Isolated From the Porcine Gut Modulate Innate Immune Responses in Epithelial Cells and Improve Protection Against Intestinal Viral-Bacterial Superinfection
Source: Front Immunol. 2021 Jun 7;12:652923. doi: 10.3389/fimmu.2021.652923 (PMC8215365; doi:10.3389/fimmu.2021.652923)
Supplement: Supplementary file 8 [file Table_1.docx]

**Supplementary Table 1.** Primer sequences used in this study.

| \| **Gene** \| **Sense primer** \| **Antisense primer** \|  \| \| --- \| --- \| --- \| --- \| \| pβ-actin \| TGG ATA AGC TGC AGT CAC AG \| GCG TAG AGG TCC TCC CTG ATG T \|  \| \| pIFN-β \| AGTTGCCTGGGACTCCTCAA \| CCTCAGGGACCTCAAAGTTCAT \|  \| \| pIFN-λ \| CCTTAGAGGCTGAGCTAGACTTGAC \| AGCCTGAAGTTCGACGTGGATG \|  \| \| pMxA \| GAG GTG GAC CCC GAA GGA \| CAC CAG ATC CGG CTT CGT \|  \| \| pRNaseL \| GCAGCCGAGCCAACGATA \| AGCTCCCGTCGCTCTCACT \|  \| \| pPKR \| CCCTGCACTTCTAGCCATCT \| CGACCACTGGCCATTTCTTTC \|  \| \| pTLR2 \| ACA TGA AGA TGA TGT GGG CC \| TAG GAG TCC TGC TCA CTG TA \|  \| \| pTLR3 \| TAGAGACATGGATTGCTCCC \| AAC TTC TGG AAT GCA GGT CC \|  \| \| pTLR4 \| CTCTGCCTTCACTACAGAGA \| CTCTGCCTTCACTACAGAGA \|  \| \| pNOD1 \| CTGTCGTCAACACCGATCCA \| CCAGTTGGTGACGCAGCTT \|  \| \| pNOD2 \| GAGCGCATCCTCTTAACTTTCG \| ACGCTCGTGATCCGTGAAC \|  \| \| pRIG-I \| TATCCGAGCAGCAGGCTTTG \| CTCGTTGCTGGGATCTATGGCC \|  \| \| pMCP-1 \| ACA GAA GAG TCA CCA GCA GCA A \| GCC CGC GAT GGT CTT G \|  \| \| pIL-6 \| TCC ATA AGC TGC AGT CAC AG \| ATT ATC CGA ATG GCC CTC AG \|  \| \| pIL-8 \| GCT CTC TGT GAG GCT GCA GTT \| TTT ATG CAC TGG CAT CGA AGT T \|  \| \| pIL-12 \| AGT TCC AGG CCA TGA ATG CA \| TGG CAC AGT CTC ACT GTT GA \|  \| \| pIL-18 \| TGAACCGGAAGACAATTGCATCAG \| CCAGGTCTTCATCGTTTTCAGCTAC \|  \| \| pTNFα \| CGA CTC AGT GCC GAG ATC AA \| CCT GCC CAG ATT CAG CAA AG \|  \| \| pA20 \| CCT CCC TGG AAA GCC AGA A \| GTG CCA CAA GCT TCC TCA CTT \|  \| \| pBCL-3 \| CGA CGC GGT GGA CAT TAA G \| ACC ATG CTA AGG CTG TTG TTT TC \|  \| \| pTollip \| TAC CGT GGG CCG TCT CA \| CCG TAG TTC TTC GCC AAC TTG \|  \| \| pIRAK-M \| TGG AGC AGC CTT GAA TCC TT \| TGG ATA ACA CGT TTG GGA ATC TT \|  \| \| pMKP-1 \| AAC GAG GGT CAG GCT TTT CC \| TCC CCA ATG TGC TGA GTT CAG \|  \| \| pSIGIRR \| ATG TGA AGT GTC GGC TCA ATG T \| TTC ATC TCC ACC TCC CCA TAC T \|  \| |
| --- | --- | --- | --- | --- | --- | --- | --- | --- | --- | --- | --- | --- | --- | --- | --- | --- | --- | --- | --- | --- | --- | --- | --- | --- | --- | --- | --- | --- | --- | --- | --- | --- | --- | --- | --- | --- | --- | --- | --- | --- | --- | --- | --- | --- | --- | --- | --- | --- | --- | --- | --- | --- | --- | --- | --- | --- | --- | --- | --- | --- | --- | --- | --- | --- | --- | --- | --- | --- | --- | --- | --- | --- | --- | --- | --- | --- | --- | --- | --- | --- | --- | --- | --- | --- | --- | --- | --- | --- | --- | --- | --- | --- | --- | --- | --- | --- | --- | --- | --- | --- |
